# Variability and genetic merits of white Guinea yam landraces in Nigeria

**DOI:** 10.3389/fpls.2023.1051840

**Published:** 2023-02-06

**Authors:** Paterne A. Agre, Alex Edemodu, Jude E. Obidiegwu, Patrick Adebola, Robert Asiedu, Asrat Asfaw

**Affiliations:** ^1^ International Institute of Tropical Agriculture (IITA), Ibadan, Nigeria; ^2^ National Root Crops Research Institute, Umudike, Abia State, Nigeria; ^3^ International Institute of Tropical Agriculture (IITA), Abuja, Nigeria

**Keywords:** breeding value, DArTseq markers, dioscorea, population structure, trait profiling, variety development

## Abstract

**Introduction:**

Landraces represent a significant gene pool of African cultivated white Guinea yam diversity. They could, therefore, serve as a potential donor of important traits such as resilience to stresses as well as food quality attributes that may be useful in modern yam breeding. This study assessed the pattern of genetic variability, quantitative trait loci (QTLs), alleles, and genetic merits of landraces, which could be exploited in breeding for more sustainable yam production in Africa.

**Methods:**

A total of 86 white Guinea yam landraces representing the popular landraces in Nigeria alongside 16 elite clones were used for this study. The yam landraces were genotyped using 4,819 DArTseq SNP markers and profiled using key productivity and food quality traits.

**Results and discussion:**

Genetic population structure through admixture and hierarchical clustering methods revealed the presence of three major genetic groups. Genome-wide association scan identified thirteen SNP markers associated with five key traits, suggesting that landraces constitute a source of valuable genes for productivity and food quality traits. Further dissection of their genetic merits in yam breeding using the Genomic Prediction of Cross Performance (GPCP) allowed identifying several landraces with high crossing merit for multiple traits. Thirteen landraces were identified as potential genitors to develop segregating progenies to improve multiple traits simultaneously for desired gains in yam breeding. Results of this study provide valuable insights into the patterns and the merits of local genetic diversity which can be utilized for identifying desirable genes and alleles of interest in yam breeding for Africa.

## 1 Introduction

Yam is a monocotyledonous vine crop cultivated for the consumption of its starchy underground tubers and aerial bulbils. It belongs to the genus *Dioscorea*, the major genus within the family of Dioscoreaceae, which spreads throughout the tropics and subtropics, with ~600 species (Wilkin et al., 2005; Darkwa et al., 2020a; Sugihara et al., 2021). Of these, eleven species are mainly cultivated for food and income, while many others of wild origins are known for their bioactive compounds suitable for pharmaceutical applications (Obidiegwu et al., 2020; Darkwa et al., 2020a; Price et al., 2020; Lebot et al., 2022). Among the cultivated species, the white Guinea yam (*Dioscorea rotundata* Poir.), water yam (*Dioscorea alata* L.), and yellow yam (*Dioscorea cayenensis* Lam.) represent more than 95% of the global yam production (Lebot, 2009).

Yam plants primarily propagate through vegetative means but often flower and produce botanical seeds. Many of the flowering yam genotypes are dioecious, with male (staminate) and female (pistillate) flowers develop on separate plants, hence are highly heterozygous due to their obligate outcrossing nature (Tamiru et al., 2017; Mondo et al., 2021). Yam plants have varying ploidy levels (2n=40, 60, and 80) within and between species with basic chromosomic number x = 20 (Sugihara et al., 2021).

The cultivated yam plays an essential role in ensuring food security and improving the livelihoods of millions of people in Africa (Sanginga and Mbabu, 2015). According to FAOSTAT (2021), ~92% of global yam production came from the West African yam belt, while Nigeria alone accounted for 65.5% of the total production. In this region, yam is cash and preferred staple food crop, providing carbohydrates, essential minerals, and vitamins (Sanginga and Mbabu, 2015). It is also integral to people’s socio-cultural and religious belief systems (Lebot, 2009; Obidiegwu and Akpabio, 2017).

Landraces with different historical origins, distinct identities, values, and adaptations constitute the dominant parts of the cultivated variability exhibited by yam in West Africa (Akakpo et al., 2017). Obidiegwu et al. (2009) reported a high genetic diversity of yam across Nigeria compared to collections from Benin, Congo, Côte d’Ivoire, Equatorial Guinea, Gabon, Ghana, Sierra Leone, and Togo. Recent studies reported the presence of moderate to high landrace diversity in Benin Republic (Agre et al., 2021a) and Côte d’Ivoire (Bakayoko et al., 2021). The tradition of continued domestication from the wild relatives by African farmers contributes to the high level of varietal and genetic diversity (Mignouna and Dansi, 2003; Dumont et al., 2006; Scarcelli et al., 2006; Agre et al., 2021a; Adewumi et al., 2022). In the yam-producing areas, farmers face many constraints (pest and disease infestation, poor soil fertility, lack of access to productive varieties, underdeveloped agronomic practices, etc.) that could potentially lead to severe yield losses and rapid genetic erosion (Dansi et al., 2013; Darkwa et al., 2020a). It is, therefore, wise to systematically collect and assess available yam landrace diversity for proper maintenance and identify desirable genes and alleles of interest.

Different techniques exist for genetic diversity assessment. Of these techniques, morphological markers are routinely used to assess yam genetic diversity (Darkwa et al., 2020a). Diversity assessment using morphological markers such as size, form, and number of tubers; bulbil formation, presence of spines on the stem, twining direction, leaf shape, etc., often led to misclassification (Girma et al., 2014; Loko et al., 2017; Ude et al., 2019; Darkwa et al., 2020a). Besides, morphological markers are relatively few, display a low degree of polymorphism, and a can be influenced by the environment, hence may not provide an accurate or conclusive genetic classification of the crop (Schulman, 2007; Mulualem et al., 2018; Agre et al., 2021a).

The use of molecular markers is, an accurate method of identifying genotypes at the species level and harnessing genetic diversity in yam crop (Agre et al., 2021a; Pachakkil et al, 2021). Different molecular markers, including restriction fragment length polymorphism (RFLP) (Terauchi et al., 1992), random amplified polymorphic DNA (RAPD) (Mignouna et al., 2003; Mignouna et al, 2005), simple sequence repeat (SSR) (Mignouna et al., 2003; Loko et al., 2017; Pachakkil et al, 2021), inter-simple sequence repeat (ISSR) (Zhou et al., 2008), amplified fragment length polymorphism (AFLP) (Mignouna et al., 2003), based on the use of next-generation sequencing (Sartie et al., 2012; Girma et al., 2014; Saski et al., 2015; Akakpo et al., 2017; Agre et al., 2019; Bhattacharjee et al., 2020; Darkwa et al., 2020b; Agre et al., 2021a; Bakayoko et al., 2021) and DNA barcoding sequencing (Girma et al., 2015; Ude et al., 2019), have been used successfully in characterizing yam diversity. The above-mentioned studies assessed the genetic diversity and possible genetic evolution of yam species; few linked the pattern of genetic diversity with its genetic merit that facilitates the identification of desirable genes/alleles addressing the current and future challenges in yam cultivation in the region. Agre et al (2019; 2021a) and Darkwa et al. (2020b) employed genomic and phenomic data and identified heterotic groups, which would facilitate parents’ choice in making crosses and harnessing population heterosis in yam breeding programs (Asfaw et al., 2021). However, and only a few genomic and agronomic data exist for the popular landraces representing a significant part of the cultivated yam variability in Africa, where the predominant crop’s production occurs. Local landraces, however, harbor potential sources of genes for stress resistance, adaptation, and quality traits in many crops breeding programs (Villa et al., 2005; Ceccarelli, 2012; Mondo et al., 2022). Therefore, proper understanding of their genetic variability and merits is crucial for the efficient use, management, and conservation of yam landraces (Mignouna et al., 2005). Thus, the main objectives of this study were to assess genetic variability, quantitative trait loci linked with key agronomic and quality traits and the genetic merits of popular landraces of white Guinea yam collected from Nigeria using DArTseq SNP markers.

## 2 Materials and methods

### 2.1 Plant Materials

A panel of 86 white Guinea yam landraces collected from 10 major yam growing regions of Nigeria (**Supplementary Table 1**). During the collection, local names and origins of the materials were documented. Landraces with the same name are differentiated by the location or collection site. A total of 16 most frequently used elite clones in the International Institute of Tropical Agriculture (IITA) yam breeding program were included as controls. Details on these control elite clones are presented in **Supplementary Table 1**.

### 2.2 Field establishment and phenotypic data analysis

The collected landraces (86) alongside the elite clones (16) were evaluated in two cropping seasons in 2018 and 2019 at IITA, Ibadan, Nigeria (7°40’19.62” N, 3°91’73.13” E, 189 m above sea level). The materials were planted using an alpha lattice design with two replications and five plants per plot. A spacing of 1 m × 1 m was used between ridges and plants on ridges. The study materials were phenotyped for key traits such as tuber yield, dry matter, tuber browning (tuber flesh oxidation), yam mosaic virus resistance, and plant vigor as per procedures described in the yam crop ontology (Asfaw, 2016). Phenotypic data were analyzed to estimate the Best Linear Unbiased Estimations (BLUEs) as surrogates of genetic values using the lme4 package (Bates et al., 2010), and mean comparisons were made using the ggplot2 package (Wickham, 2016) in R statistical computing environment (R Core Team, 2019). Phenotypic correlation was conducted among the five evaluated traits using PerformanceAnalytics R package (Brian and Carl, 2020). Genotypic, environmental, and phenotypic variances, broad-sense heritability, and the genotypic and phenotypic coefficients of variations were calculated using the variability r package (Popat et al., 2020).

### 2.3 Genotypic data assessment

One gram of young, healthy, and fully expanded leaves was sampled per genotype. Deoxyribonucleic acid (DNA) was extracted from the leaf samples using the CTAB procedure with slight modification (Dellaporta et al., 1983). DNA quality and concentration were assessed through agarose gel separation (1%) and spectrophotometry using NanoDrop 2000 (Thermo Scientific). The DArTseqTM protocol for genome complexity reduction through digestion of genomic DNA and ligation of barcoded adapters was followed as described by Kilian et al. (2014). Single-read sequencing runs for 94 bases were performed to sequence libraries. Polymorphism identification, calling, and generation of quality control parameters for selecting polymorphic markers was performed in a secondary pipeline in KDCompute plug-in platform using DArTSoft14. Obtained sequences were aligned to *D. rotundata* reference genome v2 (Sugihara et al., 2020).

### 2.4 Analysis of molecular data

Hapmap file received from the DArT sequencing platform was converted into a variant call format (VCF). A total of 22,140 SNP markers were identified from the raw data. After filtering for 1% minor allele frequency (MAF), 10% missing values, and low sequence depth (<5), 4,432 SNP markers were retained for further analyses. Data were then imputed using beagle software v4.0 (Browning and Browning, 2013). Expected and observed heterozygosity were assessed for genotypes while the minor alleles frequency (MAF) was determined for the entire markers using VCFtools and PLINK. SNP distribution and density across 20 yam chromosomes were assessed using CMplot package (Yin, 2019).

Population structure analysis was performed only using genotyping data from landraces based on admixture (Alexander et al., 2009). The optimal number of clusters was inferred using k-mean analysis after varying the possible number of clusters from 2 to 10 by employing cross-validation using the Bayesian Information Criterion (BIC). Membership probabilities (MP) of each landrace in each group was estimated by implementing a 70% threshold. Landraces above that threshold were assigned to a group, while those with low MP (<70%) were considered admixed. A pairwise dissimilarity genetic distance matrix was calculated using Jaccard method implemented in the phylentropy R package (Drost, 2018). A Ward’s minimum variance hierarchical cluster dendrogram was then built from the Jaccard dissimilarity matrix using the analyses of phylogenetics and evolution (ape) package implemented in R (Paradis et al., 2004; R Core Team, 2019). Analysis of molecular variance (AMOVA) was conducted to estimate the genetic variability among and within hierarchical clusters. To assess the level of genetic diversity among and within the different state of collections, a fixation index (Fst) was calculated using Weir and Cockerham (1984) formula implemented in vcftools.

### 2.5 Genome-wide association study analysis for target traits

The association between SNP genotypes and the phenotypes was determined using two models: the K+Q and Naïve Mixed Linear Model (MLM) implemented in GAPIT (Genome Association and Prediction Integrated Tool) – R package (Lipka et al., 2012) and visualized using CMPLOT r package (Yin, 2019). The K+Q method analysis was conducted using the procedure from Yu et al. (2006) with each SNP marker considered as a fixed effect and evaluated individually through the following formular Y = Xβ + Wα + Qv + Zu + ϵ; where Y is the observed vector of means; β is the fixed effect vector (p × 1) other than molecular marker effects and population structure (from the principal component); α is the fixed effect vector of the SNP markers; ν is the fixed effect vector from the population structure; u is the random effect vector from the polygenic background effect; X, W, and Z are the incidence matrices from the associated β, α, ν, and u parameters; ϵ is the residual effect vector. To detect reliable associations, a threshold (>4) was set calculated as follows: -log10(0.05/np), where np is the number of the total SNP markers. For the Naïve, only the markers were considered in the model. The marker effect or SNP contribution was estimated for the significant SNPs using multiple regression analysis using lme4 package (Bates et al., 2010), where the trait was considered a response variable while the SNP markers above the Bonferroni threshold for the trait were used as the independent variable.

### 2.6 Estimation of genetic merit for multi-traits

Each trait was analyzed using a mixed-effect model based on the following equation as given in Butler et al., 2017: y = Xb +Zu + e, where y is an n[=∑rj = 1 (gr)] × 1 vector of the response variable, i.e., the response of the i^th^ genotype in the j^th^ repetition; *b* is a 1 × r vector of unknown and unobservable rep fixed effects; *u* is an m[= 1 × g] vector of unknown and unobservable genotype random effects; X is an n × r design matrix of 0s and 1s relating *y* to *b*; Z represents an n × r design matrix generated from marker; *e* is an n × r vector of random.

The total genetic values were used to calculate a multi-trait index based on the factor analysis and ideotype-design (FAI-BLUP) index (Rocha et al., 2018; Olivoto and Nardino, 2021). FAI-BLUP index was computed to identify the best genotypes based on multi-trait, free from multicollinearity. Plant vigor, tuber yield, oxidative browning index, tuber dry matter, and yam mosaic virus severity score were used to identify the best genotypes. A radar chart was then generated using the radarchart function implemented in metan r package (Olivoto and Dal'Col Lúcio, 2020). Weakness and strength of the analyzed landraces were visualized using a radar chart generated using the radarchart function implemented in metan r package (Olivoto and Dal'Col Lúcio, 2020).

### 2.7 Estimation of genomic prediction and cross performance

We estimated the prediction of cross performance in ASReml-R (Butler et al., 2017) using the following formula given by Falconer and Mackay. (1996) M_F1_ = a(p - q – y) + d[2pq + y(q - p)]

Where M_F1_ is the predicted mean genotypic value of the cross (F_1_), a and d are additive and dominance effect of the SNP marker, *p* and *q* represent the allele dosage in one parent and *y=pp'=q-q'* represents the gene frequency difference between two parents. The total cross merit value was then estimated for all the landraces considering them as parents. The sex of each parent was determined to eliminate cross-combinations between landraces of the same sex. It is noteworthy that *D. rotundata* is predominantly dioecious, with male and female flowers borne on separate individuals (Mondo et al., 2020).

## 3 Results

### 3.1 Phenotypic profiles of assessed genotypes

**Figure 1:**
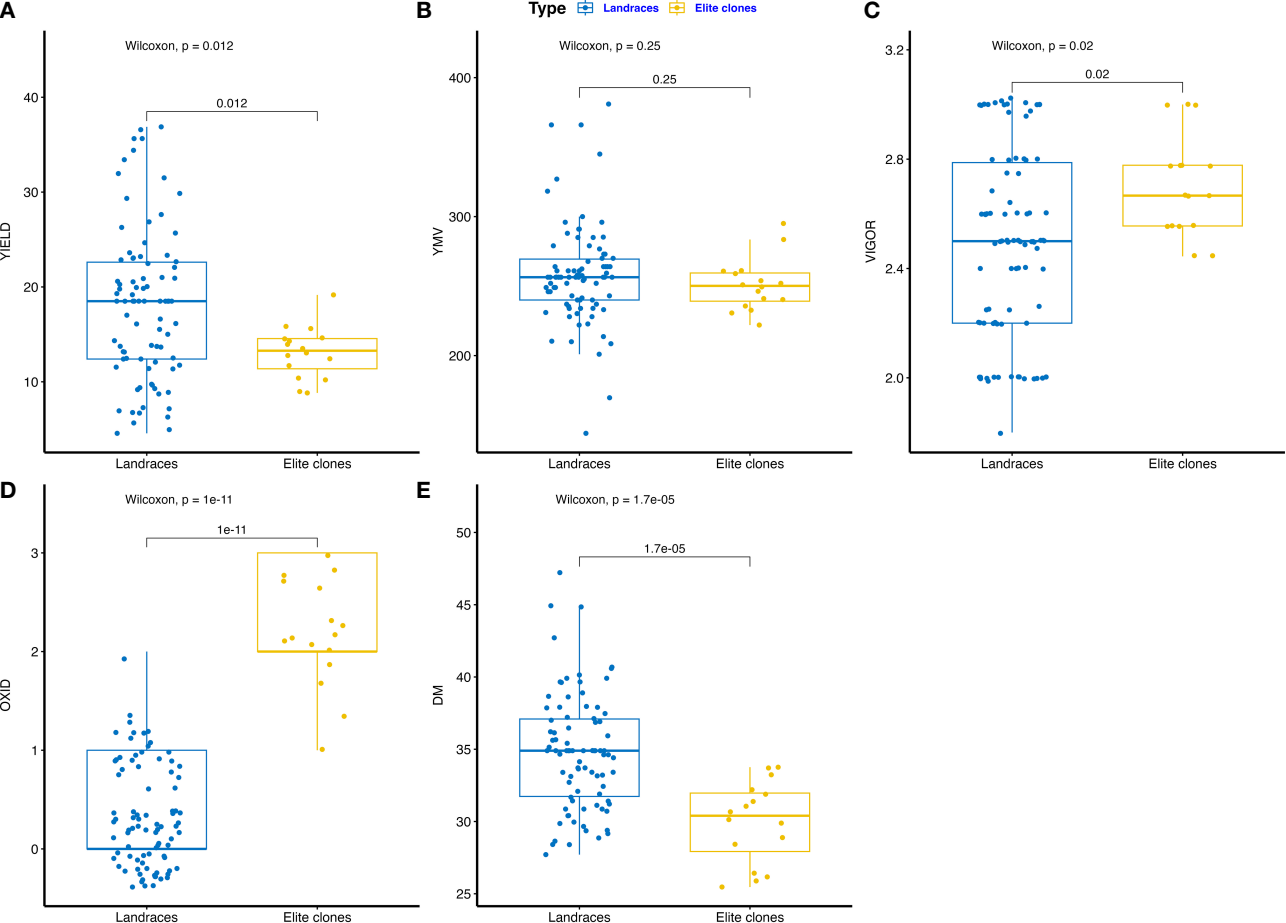
Boxplot comparing trait profile of the farmers’ landraces and elite clones from IITA yam breeding pipelines assessed: **(A)** tuber yield (t ha^-1^), **(B)** yam mosaic virus severity (AUDPC value), **(C)** plant vigor (scale), **(D)** tuber flesh oxidation (scale), **(E)** DM – tuber dry matter (%), YMV – yam mosaic virus, OXID – tuber flesh oxidation.

The phenotypic performance of farmers’ landraces and IITA elite breeding lines are presented in **Figures 1A–E** and **Supplementary Table 2**. Fresh tuber yield varied from 8.83 to 19.16 t ha^-1^ for elite clones and 4.57 to 36.88 t ha^-1^ for farmers’ landraces (**Figure 1A**). The area under the disease progression curve (AUDPC) for yam mosaic virus (YMV) severity ranged from 221.99 to 295.06 for elite clones and 144.08 to 381.00 for farmers’ landraces (**Figure 1B**). Plant vigor varied from 2.44 to 3.00 for elite clones and 1.80 to 3.02 for landraces (**Figure 1C**). Tuber oxidation scores ranged from 1.00 to 3.00 among elite clones, whereas it was 0.00 to 2.00 for landraces (**Figure 1D**). Dry matter ranged from 25.56 to 33.75% for elite clones and 27.71 to 47.22% for farmers’ landraces (**Figure 1E**). The farmers’ landraces had higher mean performance for dry matter and fresh tuber yield with less tendency of tuber flesh oxidation. Elite clones from the breeding pipeline were generally more vigorous and displayed lower AUDPC values for YMV severity scores. Phenotypic correlation analysis revealed the presence of a high and positive correlation between the fresh tuber yield and plant vigor (R = 0.69, p<0.001). The yam mosaic virus severity score displayed negative correlation with plant vigor, tuber dry matter and with the tuber oxidative browning index (**Supplementary Figure 1**).

High phenotypic variations were observed for most of the assessed traits. Broad-sense heritability ranged from 0.33 for YMV severity to 0.47 for dry matter. The narrow-sense heritability ranged from 0.29 for YMV severity to 0.41 for fresh tuber yield and dry matter (**Table 1**). The estimated breeding values of the genotypes for traits assessed are presented in **Supplementary Table 3**. For traits like dry matter, the breeding value ranged from -5.23 (Iphara) to +5.44 (Iki), while the highest breeding value for the fresh tuber yield (12.35) was observed for Miyamiyo.

**Table 1:** Genetic parameter estimates in white Guinea yam farmer landraces.

Traits	H^2^	h^2^	V_g_	V_e_	V_p_	CV_g_(%)	CV_p_(%)	Mean
Yield	0.43	0.41	31.32	19.80	51.11	53.05	67.78	10.54
YMV	0.33	0.29	1549.69	2113.47	3669.15	11.80	18.14	333.50
Vigor	0.39	0.33	0.05	0.16	0.21	7.70	16.40	2.79
Oxid	0.44	0.24	0.01	0.02	0.03	551.93	829.50	0.02
DM	0.47	0.41	7.00	2.63	9.63	8.73	10.24	30.30

Vg, genotypic variance; Ve, environmental variance; Vp, phenotypic variance; H2, broad-sense heritability; h2, narrow-sense heritability; CVg(%), genotypic coefficient of variation; CVp(%), phenotypic coefficient of variation; mean: overall experiment mean for the considered trait. Yield, tuber yield per ha; YMV, yam mosaic virus severity; Vigor, plant vigor; DM, dry matter; Oxid, tuber flesh oxidation.

### 3.2 Genetic variability and population structure of the landraces

Results from the SNP genotyping analysis on landraces are presented in **Table 2**. A total of 4,432 filtered SNP markers distributed across the 20 yam chromosomes were identified. Chromosome 13 had the least SNP markers (88), while chromosome 5 had the highest number (473) (**Supplementary Figure 2** **)**. SNP density plot revealed the presence of a relatively high SNP density in the telomeric region across the 20 chromosomes. Observed heterozygosity (Ho) varied from 0.113 (on chromosome 17) to 0.173 (on chromosome 3), with an average of 0.141. The expected heterozygosity (He) ranged between 0.210 (on chromosome 1) and 0.257 (on chromosome 8), with a mean of 0.234. The minor allele frequency (MAF) ranged between 0.134 (on chromosome 1) and 0.170 (on chromosome 8), with an average of 0.151. Polymorphic information content (PIC) varied from 0.232 (on chromosome 1) to 0.265 (on chromosome 16), with a mean value of 0.247. Gene diversity ranged from 0.259 (on chromosome 1) to 0.297 (on chromosome 16), with an average of 0.275 (**Table 2**).

**Table 2:** Summary statistics of SNP markers across 20 white Guinea yam chromosomes.

Chromosome	No. of SNPs	Ho	He	MAF	PIC	Gene diversity
Chr1	133	0.147	0.210	0.134	0.232	0.259
Chr2	141	0.129	0.236	0.148	0.245	0.275
Chr3	144	0.173	0.230	0.149	0.254	0.284
Chr4	352	0.119	0.248	0.161	0.241	0.268
Chr5	473	0.131	0.245	0.158	0.253	0.282
Chr6	177	0.139	0.221	0.139	0.237	0.265
Chr7	267	0.134	0.221	0.140	0.239	0.264
Chr8	287	0.129	0.257	0.170	0.253	0.282
Chr9	127	0.143	0.229	0.147	0.238	0.265
Chr10	180	0.152	0.224	0.143	0.255	0.286
Chr11	177	0.146	0.220	0.140	0.234	0.260
Chr12	179	0.146	0.214	0.136	0.234	0.260
Chr13	88	0.156	0.245	0.161	0.239	0.268
Chr14	253	0.154	0.228	0.149	0.260	0.290
Chr15	227	0.127	0.256	0.167	0.253	0.283
Chr16	176	0.162	0.235	0.153	0.265	0.297
Chr17	225	0.113	0.243	0.156	0.248	0.276
Chr18	224	0.133	0.240	0.155	0.260	0.290
Chr19	441	0.124	0.246	0.160	0.245	0.272
Chr20	161	0.165	0.227	0.149	0.247	0.275
Total	4432					
Average	221.6	0.141	0.234	0.151	0.247	0.275

Chr, chromosome; Ho, observed heterozygosity; He, expected heterozygosity; MAF, minor allele frequency; PIC, polymorphic information contest.

**Figure 2:**
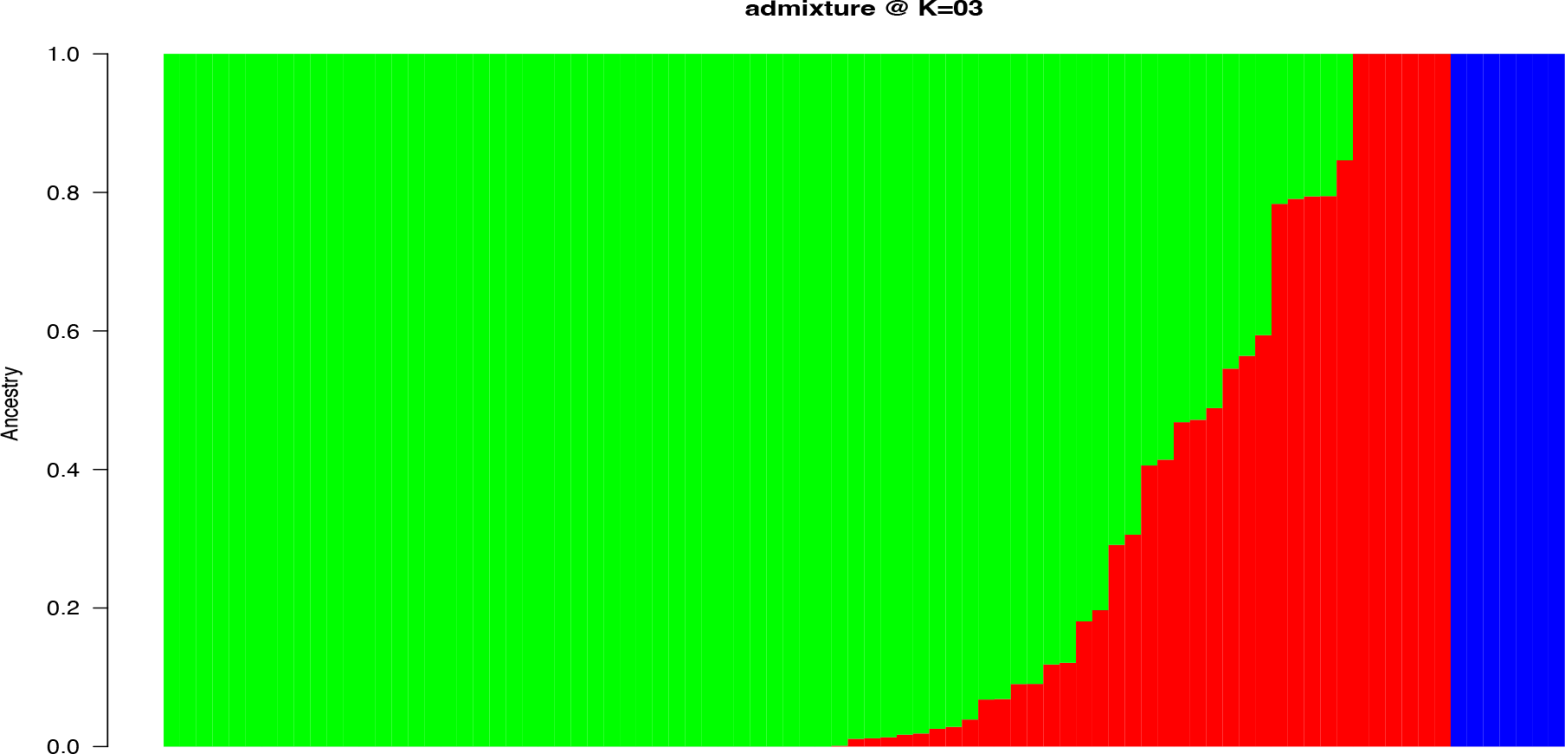
Population structure with K = 3 for 86 yam farmers’ landraces from Nigeria using 4,432 high-quality SNPs. The landraces represented by vertical bars along the horizontal axis were classified into *K* color segments based on their estimated membership fraction in each *K* cluster. Clusters (1, 2, and 3) are represented by blue, red, and green colors, respectively.

Population structure-based Bayesian Information Criteria (BIC) showed a rapid elbow at *k* = 3 and was used as the optimum number of clusters (**Supplementary Figure 3**). The cluster 3 (green) had the highest proportion of accessions (71%) followed by cluster 2 (red) (14%) and 1(blue) (8%) (**Figure 2**). Six of the characterized landraces were admixtures as they had assignment probabilities below 0.7 and could not, therefore, be assigned to any specific group (**Supplementary Table 4**). In the cluster 3, members were represented by landraces collected from different parts of Nigeria, while those in cluster 1 were mainly from eastern Nigeria.

**Figure 3:**
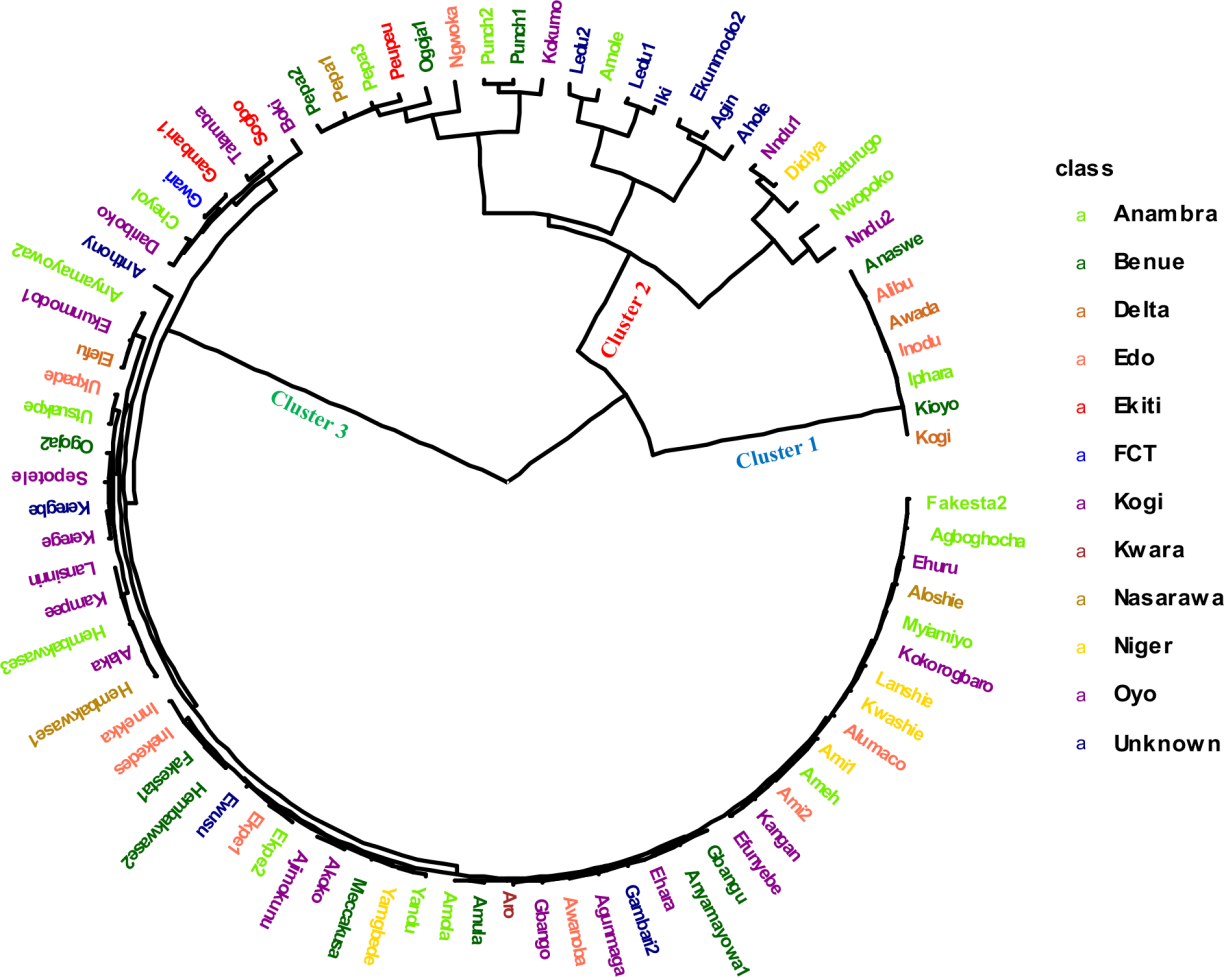
Hierarchical clustering analysis based on 4,432 DArT-SNP markers showing the genetic relationship among 86 Nigerian white Guinea yam landraces. The landraces collected from different locations of Nigeria are presented in different color.

The genetic distance between the landraces was computed as 1 - IBS (identity by state), with IBS defined as the probability that alleles drawn at random from two individuals at the same locus are the same. The grouping pattern based on the IBS clustered the 86 white yam farmers’ landraces into three clusters (**Figure 3**). The cluster size varied among identified groups: cluster 1 contained 7 landraces (8%), cluster 2 had 14 landraces (16%) while cluster 3 was the largest with 65 landraces (76%). The clustering pattern of the landrace exhibited non-parallel patterns of geographic variations. Furthermore, the Fst values among the different locations showed lack of genetic subdivision between landraces from different locations (**Supplementary Table 5**).

A pairwise dissimilarity genetic distance matrix revealed that the genetic distance for the entire landraces ranged from 0.0073 to 0.4015. The two landraces that displayed the lowest genetic distance had the same name (Punch) but were collected from different locations and hence considered as the same genetic material. In cluster 1, the lowest genetic distance was 0.0225, while the highest distance was 0.053. Landraces in this cluster had relatively higher fresh tuber yield, lower AUDPC values for YMV disease severity scores, and rough tubers (**Table 3**). Landraces in cluster 2 were characterized by higher AUDPC values for YMV disease severity scores and smooth tuber texture (**Table 3**). In cluster 3, the lowest genetic distance (0.0073) was recorded between several landraces (**Figure 3**). Landraces in cluster 3 were characterized by lower yield but higher tuber dry matter content and slightly rough tubers (**Table 3**).

**Table 3:** Agronomic performance of farmers’ landraces and genetic characteristics of clusters generated using SNP markers.

Variables	Cluster 1 (7)	Cluster 2 (14)	Cluster 3 (65)
Tuber yield (t ha^-1^)	19.21	18.41	17.60
YMV severity (AUDPC value)	218.48	272.12	258.77
Dry matter (%)	34.22	34.32	35.00
Tuber oxidation	Non-oxidation	Non-oxidation	Non-oxidation
Plant vigor	High	Medium	Medium
Tuber texture	Slightly rough	Smooth	Slightly rough
Flesh color	White	White	White
Average GD	0.23	0.18	0.36
Average He	0.29	0.39	0.33
Average Ho	0.32	0.40	0.36

The numbers in the brackets represent the number of members in a cluster; YMV, yam mosaic virus; He, expected heterozygosity; Ho, observed heterozygosity; GD, genetic distance.

Classification based on admixture and hierarchical clustering (HC) methods agreed in assigning most of the landraces (**Supplementary Table 4**). The six landraces considered as admixed (by the admixture clustering method) were fully assigned by the hierarchical clustering method to clusters 2 and 3, with three landraces each. The AMOVA revealed high genetic variability (71%) among hierarchical clusters, while the genetic variability was low within clusters (**Table 4**).

**Table 4:** Analysis of molecular variance (AMOVA) among and within white Guinea yam farmers’ landraces from Nigeria.

Sources of variation	df	SS	MS	Est. Var.	% variation
Among clusters	2	31997.92	15998.96	918.68	71
Within clusters	83	30766.93	370.69	370.69	29
Total	85	62764.85	16369.65	1289.37	100

df, Degrees of freedom; SS, sum of squares; MS, mean squares; Est.Var, estimated variance; % variation, percentage contribution to the total variability.

### 3.3 Genome-wide association results for key traits

**Figure 4:**
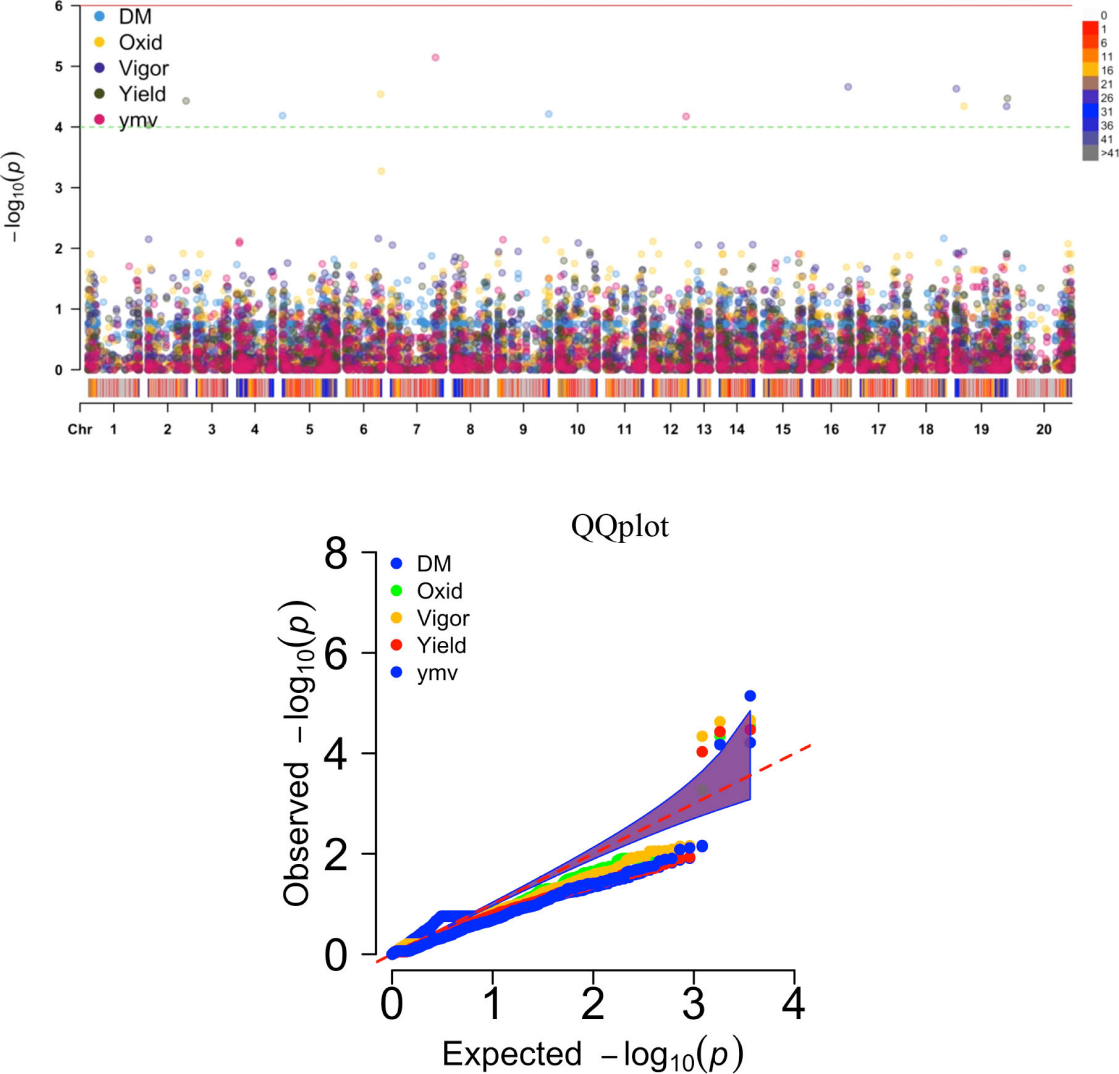
Manhattan and QQ plots displaying regions of genome significantly associated with natural variation for five traits targeted in this study using the K+Q model. DM, dry matter (%); Oxid, tuber flesh oxidation (scale); Vigor, plant vigor (scale); Yield, tuber yield (t ha-1); ymv, yam mosaic virus severity (AUDPC value).

Thirteen SNP markers were tightly associated with the five studied traits (**Table 5**, **Figure 4**). Three SNP markers on three chromosomes were identified as associated with the tuber dry matter content. The associated SNP markers displayed high total phenotypic variance >21% with a LOD score above 4. Four SNP markers were associated with the tuber browning index (tuber flesh oxidation). The four markers explained high total phenotypic variance with positive SNP marker effect except the marker located on chromosome 5. A single SNP marker on chromosome 19 was linked with the plant vigor. The marker explained a high phenotypic variance of 28.53% with a positive marker effect and LOD score above 4. For the fresh tuber yield, three SNP markers were identified on two chromosomes, 1 & 19. Two SNP markers on chromosome 19 displayed a positive value as marker effect with a total explained phenotypic variance of 8.38 and 11.82%. The SNP marker located on chromosome 1 showed a negative marker effect with the highest LOD score. For the YMV severity score, three SNP markers were identified on chromosomes 7, 9 & 15. The three markers linked with the YMV displayed high R^2^ and LOD scores above 4. Notably, the region of chromosome 19 was identified as linked with four of the traits analyzed. Also, the SNP marker “chr19_5210667” was associated with yield and plant vigor. Using the naïve analysis, no significant QTL was detected above the suggestive threshold(**Supplementary Figure 4**).

**Table 5:** SNP markers associated with target traits.

Trait	SNP	Chr	Pos (bp)	P-value	MAF	Effect	LOD	R^2^(%)
YMV	chr7_26854456	7	26854456	3.4727E-06	0.15	113.15	5.46	26.50
	chr9_3326055	9	3326055	2.3473E-05	0.15	113.15	4.63	26.50
	chr15_22775177	15	22775177	1.5449E-05	0.06	-27.92	4.81	21.01
Yield	chr19_5210667	19	5210667	7.27E-05	0.19	6.19	4.14	11.82
	chr1_2096240	1	2096240	1.43E-05	0.15	-8.43	4.84	10.43
	chr19_30713072	19	30713072	3.04E-05	0.14	8.09	4.52	8.38
Vigor	chr19_5210667	19	5210667	4.04E-05	0.19	0.37	4.39	28.53
Oxid	chr16_21103453	16	21103453	1.06E-05	0.07	1.36	4.97	21.52
	chr9_28870658	9	28870658	1.2548E-05	0.08	1.06	4.90	15.37
	chr19_30315263	19	30315263	1.3358E-05	0.05	0.89	4.87	15.27
	chr5_18430863	5	18430863	1.9022E-05	0.13	-0.46	4.72	7.82
DM	chr9_30457800	9	30457800	1.1828E-05	0.06	12.09	4.93	14.47
	chr14_5786575	14	5786575	2.7739E-05	0.42	-2.43	4.56	14.30
	chr19_30758641	19	30758641	9.1471E-05	0.07	3.61	4.04	13.76

Chr, chromosome; bp, base pair; LOD, logarithm of odd score; R^2^(%), total explained phenotypic variance; MAF, minor allele frequency; DM, dry matter; Oxid, tuber flesh oxidation; YMV, yam mosaic virus.

### 3.4 Genetic merits and cross performance of the landraces

The exploratory factor analysis identified the first three factors (FA) (Eigenvalue >1) that explained 74.2% of the total variation among the traits as most discriminative. The communality after the varimax rotation of each trait’s variance explained by the three factors ranged from 0.518 for vigor to 0.843 for tuber oxidative browning. The five traits were grouped into three based on their highest genetic correlations for the first three factors (**Supplementary Table 6**). The high genetic correlation for the FA1 was observed with plant vigor, fresh tuber yield and yam mosaic virus severity score while for the FA2 with dry matter content and for the FA3 with tuber browning index (tuber flesh oxidation).

**Figure 5:**
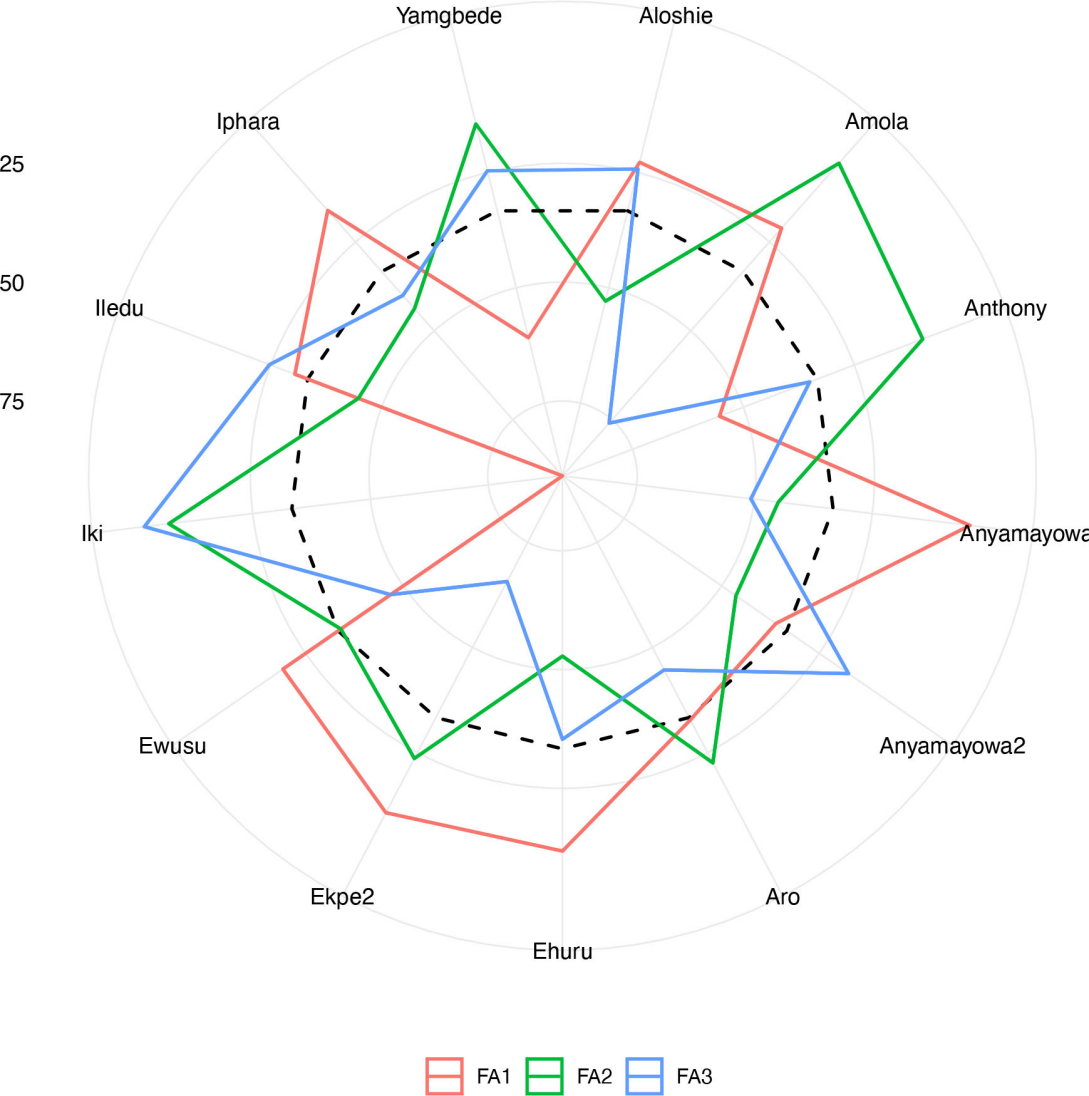
Radar chart displaying the strengths and weaknesses of selected Nigerian white Guinea yam landraces based on the FAI‐BLUP index. The dashed line shows the theoretical value if all three factors had contributed equally.

The analysis of the FAI-BLUP index ranged from 2.76 to 8.11 (**Supplementary Figure 5**). From the 86 landraces evaluated, 13 landraces with < 3.5 FAI-BLUP index values were selected as top-ranking for their high multi-trait performance (**Supplementary Figure 6** **)**. However, the predicted selection gain was in desired direction for three traits out the five. The strengths and weaknesses of the selected 13 yam landraces were presented in a radar plot which accounted by the proportion of each factor to the FAI-BLUP index of the landraces (**Figure 5**). The first factor (FA1) had the smallest contribution for eight genotypes (Ewusu, Ekpe2, Ehuru, Anyamayowa1, Amola, Aloshie, Iphara and Iledu) and highest contribution to the FAI-BLUP index of landrace Iki. Of these eight landraces, Ehuru, Aloshie and Anyamayowa1 had the most desired breeding values for plant vigor, tuber yield and YMV resistance (**Supplementary Table 3**). On other hand, Ike had undesirable breeding value for tuber yield and vigor but good for YMV resistance. Likewise, FA2 had the smallest contribution for FAI-BLUP index values of Amola, Aru, Ekpe2, Iki and Yamgbede which expressed desirable breeding values for tuber dry matter content. The third factor (FA3) had the smallest contribution for five landraces (Iki, Iledu, Yamgbede, Aloshie and Anyamayowa2) suggesting these landraces expressing low tuber browning index.

Further analysis for the genomic prediction of cross-performance of the 61 landraces that produced flowers (25 females and 36 males) generated net crossing merit for 937 cross combinations that ranged from -2.04 to 2.4 (**Supplementary Figure 7**). The 15 genotypes that didn’t flower were excluded from the crossing merit prediction. Among the 25 female flowering landraces predicted for cross performance, Bokipepa, Yandu, Meccakusa and Pepa1 showed highest crossing compatibility with 31, 31, 27, and 26 male flowering landraces, respectively (**Supplementary Table 7**). Among the male flowering landraces, Iledu, Didiya, Amola, Ekummodu, and Kioyo expressed highest cross-compatibility. The highest average crossing merit (1.68) was predicted for Pepa3 a female flowering landrace and the lowest average crossing merit -1.32 was for Ehuru, a male flowering landrace

Of the thirteen landraces identified as top ranking based on FAI-BLUP index, eight had positive net crossing merit while five had negative net crossing merit (**Supplementary Table 7**). The specific crossing merit among these 13 landraces presented in **Table 6**. The female flowering genotype Aloshie has positive specific crossing merit with Amola, Aro and Ehuru. The other female landrace Anyamayowa1 has positive specific crossing merit with Ehuru, Ewusu, Iledu and Yamgbeda while Anyamayowa2 has positive predicted crossing merit with Anthony, Aro, Ehuru, Ewusu and Yamgbede. Likewise, Ekep2 has a positive predicted specific crossing merit with Anthony, Ehuru, Iki and Yamgbede. Iphara has positive specific crossing merit with Anthony and Aro only.

**Table 6:** Genomic prediction cross performance among the 13 top ranking landraces for multi-trait performance based on FAI-BLUP index.

	Male							
Female	Amola	Anthony	Aro	Ehuru	Ewusu	Iki	Iledu	Yamgbede
Aloshie	0.20		0.16	0.04			-0.16	
Anyamayowa1			-1.36	4.28	2.32		2.2	1.72
Anyamayowa2		1.56	1.52	3.12	1.32			0.62
Ekpe2		1.32			1.16	1.04		1.22
Iphara		0.34	0.28	-0.48				-0.62

## 4 Discussion

In this study, we used DArT-SNP markers to assess the genetic diversity of yam landraces in Nigeria to identify potential sources of genes for broadening the genetic variation in yam breeding. The DArTseq genotyping detected 4,432 informative SNPs, which were unequally distributed among and within the 20 yam chromosomes. The SNP density was relatively high in the telomeric regions compared to the peri-centromeric areas. Distal euchromatin regions (telomeric regions) were more densely covered with genes and had higher recombination rates than peri-centromeric heterochromatin regions. These results in line with those of Sugihara et al. (2020). Through structure and phylogenetic tree analyses, the results displayed a non-random distribution of alleles and genotypes. The 86 yam landraces were classified into three groups through the population structure and hierarchical clustering methods. There was high correspondence in clustering patterns between the two grouping methods. Agre et al. (2019) reported a similar trend in a genetic diversity study involving 100 genotypes of *D. alata*.

Results of the AMOVA revealed high genetic variation among the three clusters and lower genetic variation among landraces within clusters. Similarly, Bakayoko et al. (2021) reported low molecular variability within groups and high between groups of *D. alata* accessions from Côte d’Ivoire using SNP markers. High levels of genetic variation among clusters of yam landraces in Nigeria indicated a lack of gene flow, possibly due to low seed-yam (mini or small whole tubers or portion of tubers used for propagation) exchange among farmers in geographically distant areas. In contrast, the low variation within cluster revealed a low degree of genetic differentiation, which may be attributed to regional preferences for some dominant varieties (Bakayoko et al., 2021). As stated by Stuart et al. (2021), sharing (small amounts) of seed-yam tubers as a gift is a common practice among farmers of the same and or different communities.

Yam breeding programs usually focus on developing and deploying superior varieties, which combine traits preferred for production and consumption (Darkwa et al., 2020a). Breeding efforts for the past five decades have resulted in breeding for superior yam varieties with high tuber yield, tolerant to pests and diseases with high tuber quality attribute which has translated into the release of several improved varieties (Darkwa et al., 2020a; Agre et al., 2022). However, genetic improvement of traits such as dry matter content and tuber browning is still a challenge in yam breeding. These traits are essential quality attributes that determine acceptability in newly developed yam varieties (Gatarira et al., 2020). Through traits profiling, we have identified landraces with high tuber yield, dry matter content, and slow rate of tuber flesh oxidation in the Nigerian farmers’ landraces. Hence, landraces with high crossing merit values for multiple traits identified in this study could be used for trait introgression by breeding programs to complement the superior characteristics (e.g., resistance to viruses and high vigor) of elite clones and to broaden the genetic variation in breeding materials for increased genetic gain.

Moreover, the SNP markers associated with natural variation for the studied traits identified herewith would be valuable resources to enhance genetic gain in yam breeding. Using a mixed linear model, we identified thirteen SNP markers linked with the five key traits in yam breeding. The same QTL region previously reported by Gatarira et al. (2020) on chromosomes 19 and 5 were identified for the DM and tuber flesh oxidation index. Likewise, many SNP markers significantly associated with variation for the tuber yield and yam mosaic virus resistance were detected. Among flanking regions in the genome for tuber yield and YMV resistance, Agre et al. (2021b) reported chromosomes 19 and 15 as the potential regions controlling tuber yield per plant and yam mosaic virus resistance, respectively, in *D. rotundata*. On chromosome 19, the same SNP location was found to control tuber yield and plant vigor (**Table 5** and **Figure 4**). Plant vigor and tuber yield displayed a positive and highly significant correlation (0.69, p<0.001). Gao et al. (2016) reported that such region should be investigated to elucidate the potentiality of developing a single SNP marker for multiple trait prediction.

In additional to the previous QTL reported by Agre et al. (2021b); Gatarira et al. (2020); Bredeson et al. (2022); Ehounou et al. (2022) the QTL identified in this study provided information on the chromosomal regions controlling yam productivity and food quality, which can be useful genomic resource information to the yam breeding community. However, these QTLs have not yet been fully utilized in the yam breeding for molecular assisted selection due to many factors, such as limited marker-trait association, small phenotypic variance explained, differences in the genetic backgrounds, and environmental effects. Previous results from traits association including linkage mapping should be investigated through meta-QTL analysis to refine the number and position of the QTLs to identify stable QTLs for marker assisted selection.

## 5 Conclusions

Population structure and hierarchical clustering methods classified the yam landraces from Nigeria into three distinct genetic groups. The AMOVA revealed higher variability among clusters than within clusters. The wide genetic variability among the Nigerian yam landraces implied that these could serve as valuable sources of novel genes for yam breeding and variety development. The promising farmers’ landraces identified with good attributes and high crossing merit values could be exploited for genetic improvement in yam breeding programs, particularly for the introgression of genes controlling high tuber yield and dry matter content and reduced tuber flesh oxidation into IITA yam breeding lines. This will translate into new, improved yam varieties with huge food security implications.

## Data availability statement

The datasets presented in this study can be found in online repositories. The names of the repository/repositories and accession number(s) can be found below: https://figshare.com/account/projects/149629/articles/21195358.

## Author contributions

AA conceptualized the study idea. PA, JEO, AA, and AE assembled the genetic materials. AA, AE, and PAA coordinated data generation. PAA analyzed the data and wrote the first draft with inputs from AA. All authors contributed to the article and approved the submitted version.
