# Supplementary material for: Variability and genetic merits of white Guinea yam landraces in Nigeria
Source: Front Plant Sci. 2023 Feb 6;14:1051840. doi: 10.3389/fpls.2023.1051840 (PMC9940711; doi:10.3389/fpls.2023.1051840)
Supplement: Supplementary file 1 [file DataSheet_1.doc]

**Supplementary files**

**
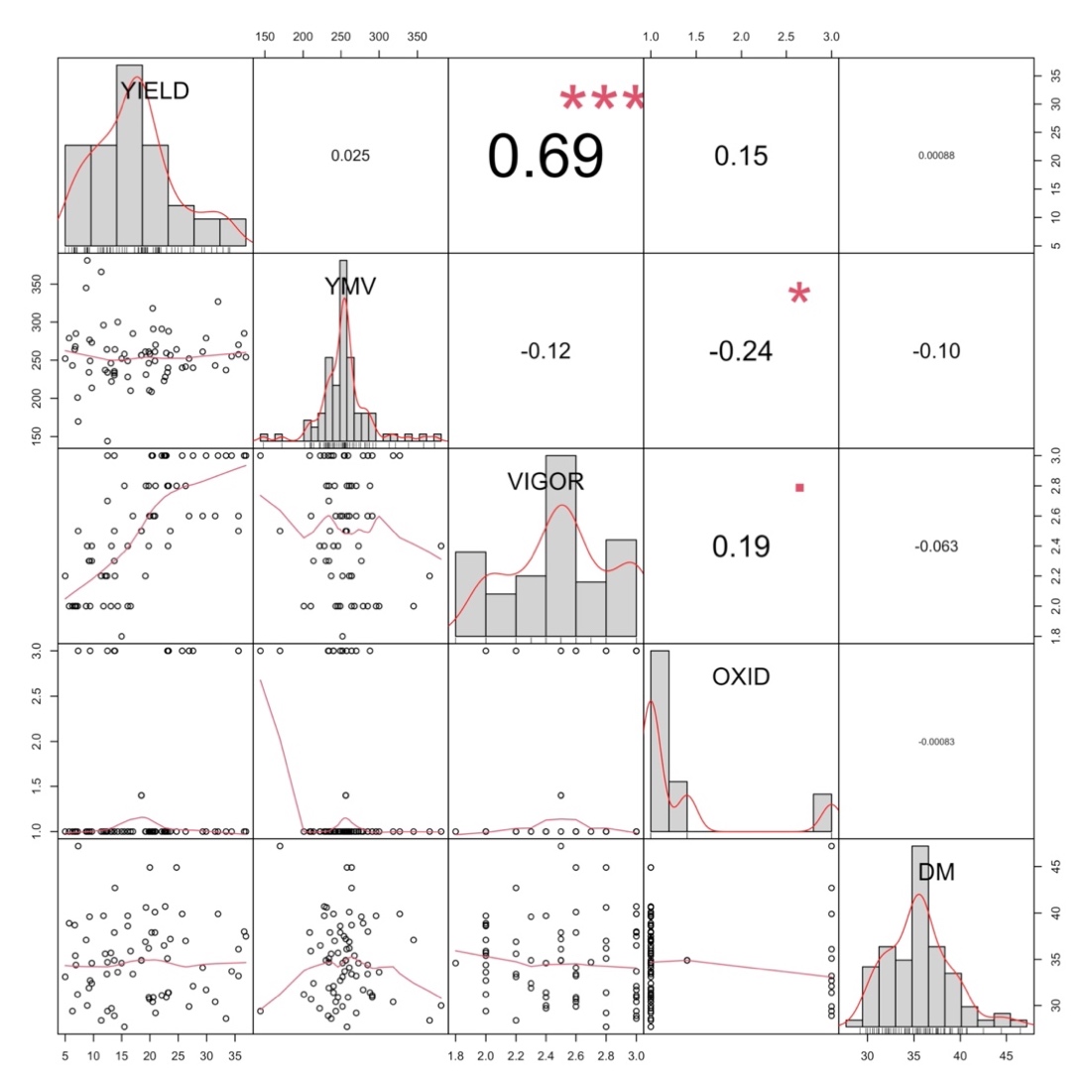
**

SUPPLEMETARY FIGURE 1 | **Correlation coefficients among five yam key agronomic and food quality traits.**


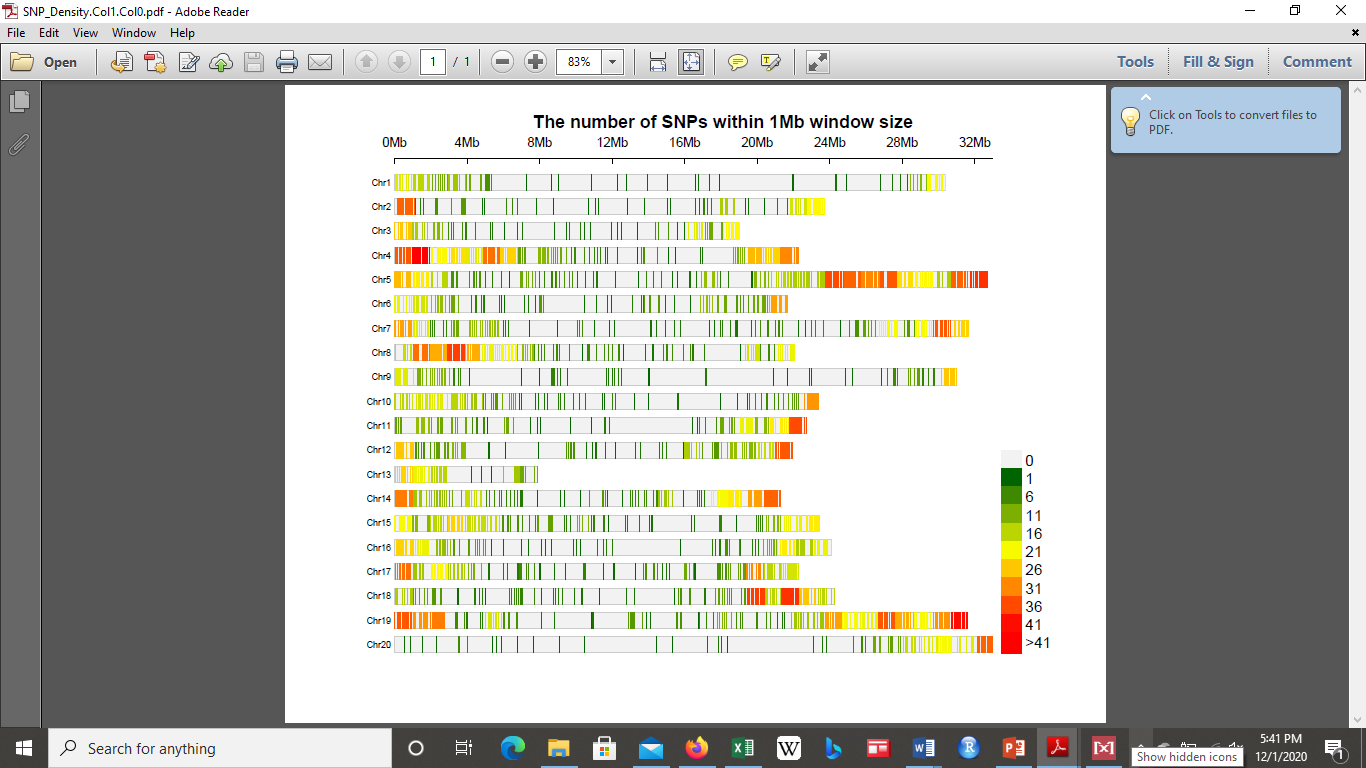


SUPPLEMENTARY FIGURE 2 | **Distribution and density of 4,432 filtered SNP markers across 20 white yam chromosomes.**


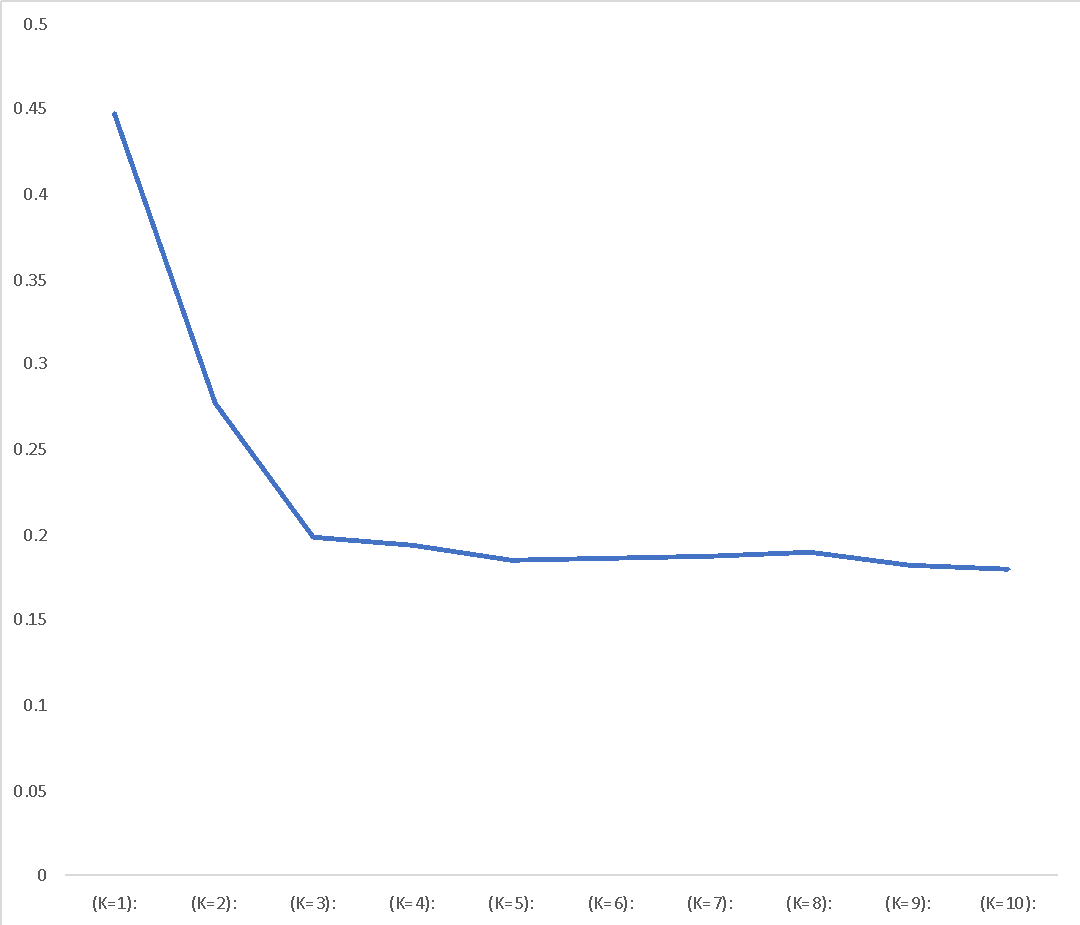


SUPPLEMETARY FIGURE 3 | **Variation of cross validation errors as a function of the number of clusters.** In the x-axis, a different number of clusters that could be considered.


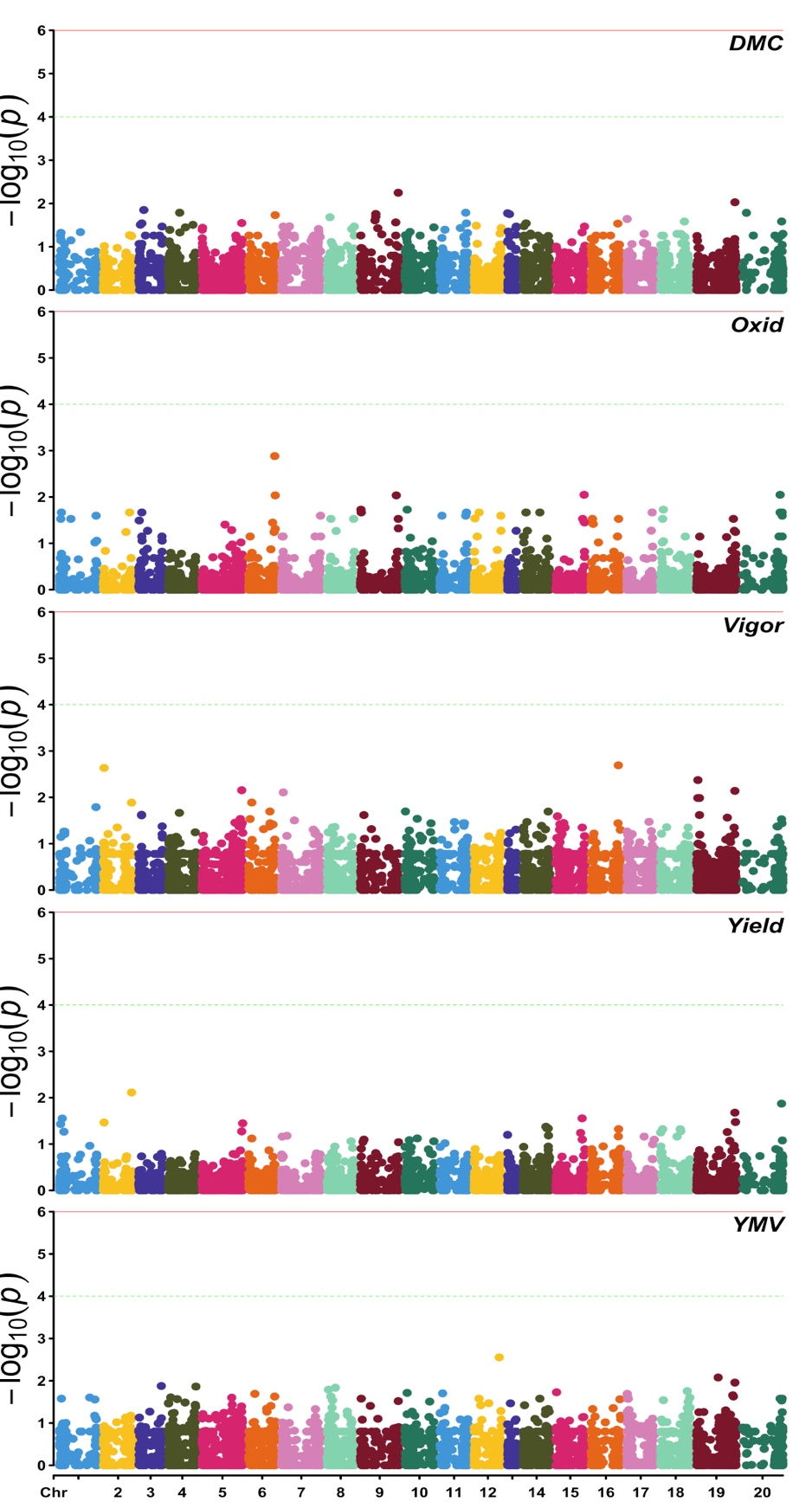


SUPPLEMENTARY FIGURE 4 | **Manhattan plot using Naïve genetic model**

**
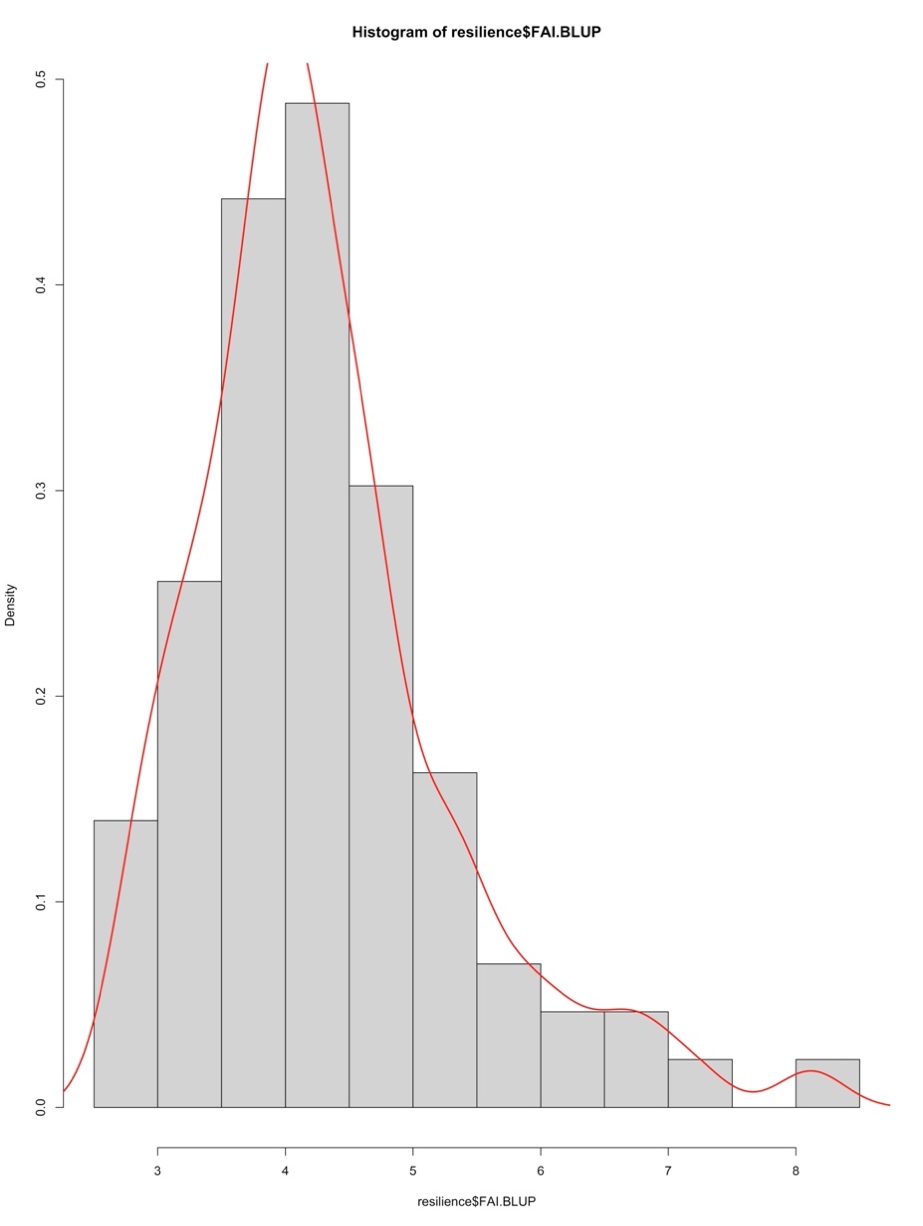
**

SUPPLEMENTARY FIGURE 5 **|** **Density plot showing the distribution of the BLUP-FAI index for the 86 yam landraces.**


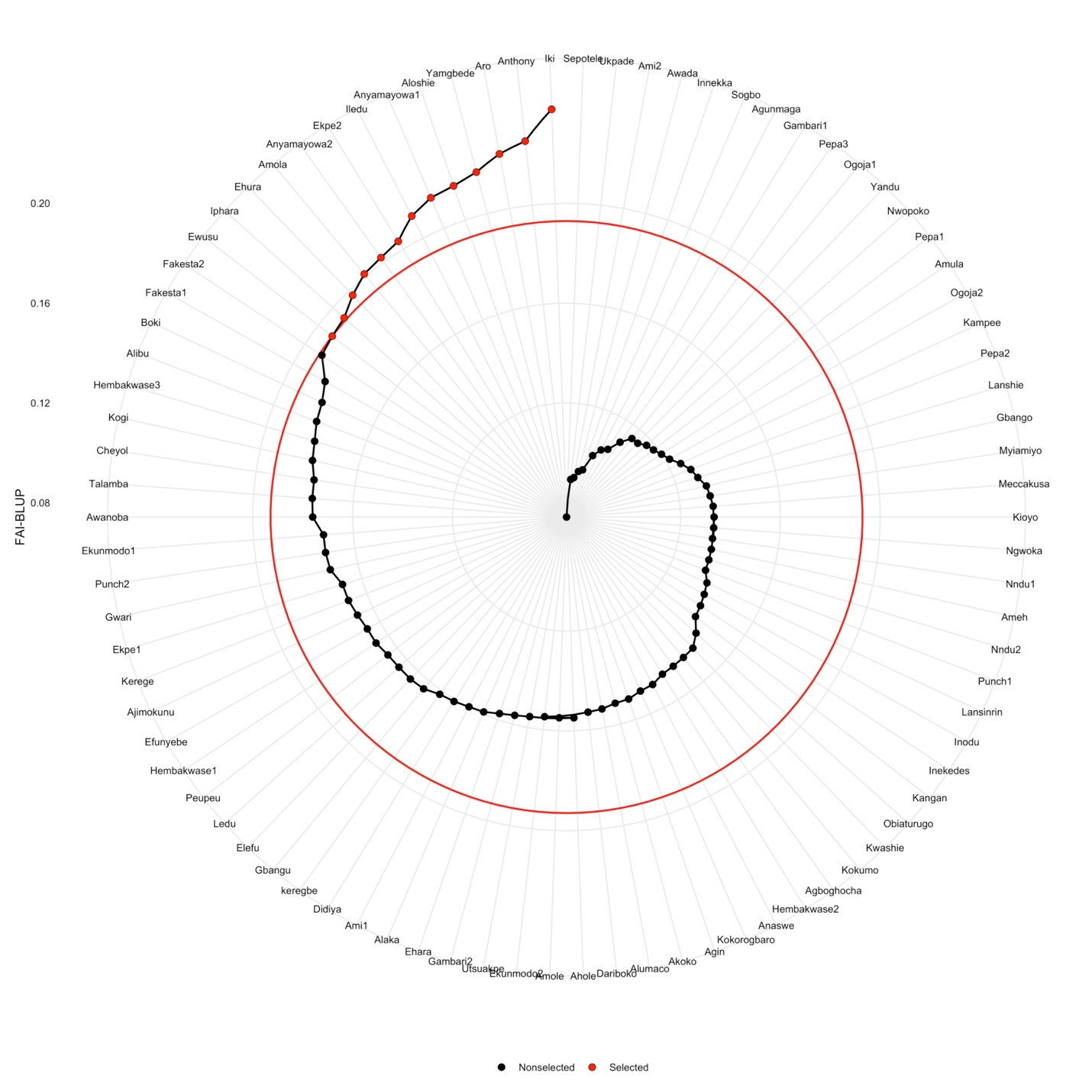


SUPPLEMENTARY FIGURE 6 | **Factor analysis and ideotype-design (FAI-BLUP) index showing the ranking and the selected yam landraces.** The selected genotypes are shown in red and the unselected in black circles.


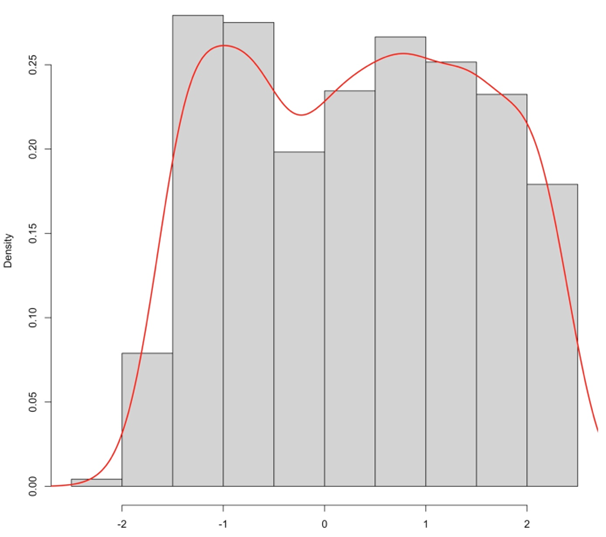


SUPPLEMENTARY FIGURE 7 | **Density plot showing the distribution of the crossing merit values for the yam landraces.**

SUPPLEMENTARY TABLE 1| **List of the 86 yam landraces and 16 breeding lines used for the study.**

| **S/N** | **Local Name** | **Village** | **LGA**^ɠ^ | **State** | **Germplasm type** |
| --- | --- | --- | --- | --- | --- |
| 1 | Alibu | Ewohimi | Esan South | Edo | Landrace |
| 2 | Anaswe | Buruku | Buruku | Benue | Landrace |
| 3 | Awada | Idumuje-Ugboko | Aniocha North | Delta | Landrace |
| 4 | Inodu | Ayogwuri | Etsako West | Edo | Landrace |
| 5 | Iphara |  | Ikwuano | Anambra | Landrace |
| 6 | Kioyo | Buruku | Buruku | Benue | Landrace |
| 7 | Kogi | Idumuje-Ugboko | Aniocha North | Delta | Landrace |
| 8 | Didiya | Gunu | Shiroro | Niger | Landrace |
| 9 | Nndu1 | Igboho | Orilope | Oyo | Landrace |
| 10 | Nndu2 | Igbope | Orilope | Oyo | Landrace |
| 11 | Nwopoko |  | Ikwuano | Anambra | Landrace |
| 12 | Obiaturugo |  | Ikwuano | Anambra | Landrace |
| 13 | Ogoja | Gboko | Gboko | Benue | Landrace |
| 14 | Pepa1 | Agyaharagu | Lafia | Nasarawa | Landrace |
| 15 | Pepa2 | Gboko | Gboko | Benue | Landrace |
| 16 | Pepa3 |  | Ikwuano | Anambra | Landrace |
| 17 | Peupeu | Iye-ekiti | Ilejemje | Ekiti | Landrace |
| 18 | Ngwoka | Ewohimi | Esan South | Edo | Landrace |
| 19 | Kokumo | Oje-Owode | Saki East | Oyo | Landrace |
| 20 | Punch1 | Gboko | Gboko | Benue | Landrace |
| 21 | Punch2 |  | Ikwuano | Anambra | Landrace |
| 22 | Amole |  | Ikwuano | Anambra | Landrace |
| 23 | Agboghocha |  | Ikwuano | Anambra | Landrace |
| 24 | Agin |  |  |  | Landrace |
| 25 | Agunmaga | Kaba | Kabba/Bubu | Kogi | Landrace |
| 26 | Ahole |  |  |  | Landrace |
| 27 | Ajimokunu | Kaba | Kabba/Bubu | Kogi | Landrace |
| 28 | Akoko | Oje-Owode | Saki East | Oyo | Landrace |
| 29 | Alaka | Kaba | Kabba/Bubu | Kogi | Landrace |
| 30 | Aloshie | Agyaragu | Lafia | Nasarawa | Landrace |
| 31 | Alumaco | Ilushi | Akoko Edo | Edo | Landrace |
| 32 | Ameh |  | Ikwuano | Anambra | Landrace |
| 33 | Ami1 | Garatu | Bosso | Niger | Landrace |
| 34 | Ami2 | Ayogwuri | Etsako West | Edo | Landrace |
| 35 | Amola |  | Ikwuano | Anambra | Landrace |
| 36 | Amula | Gboko | Gboko | Benue | Landrace |
| 37 | Anthony |  |  |  | Landrace |
| 38 | Anyamayowa1 | Gboko | Gboko | Benue | Landrace |
| 39 | Anyamayowa2 | NRCRI | Ikwuano | Anambra | Landrace |
| 40 | Aro | Ileshabaruba | Baruten | Kwara | Landrace |
| 41 | Awanoba | Ayogwuri | Etsako West | Edo | Landrace |
| 42 | Boki | Igbope | Orilope | Oyo | Landrace |
| 43 | Cheyol |  | Ikwuano | Anambra | Landrace |
| 44 | Dariboko | Kisi | Irepo | Oyo | Landrace |
| 45 | Efunyebe | Oje-Owode | Saki East | Oyo | Landrace |
| 46 | Ehara | Kaba | Kabba/Bubu | Kogi | Landrace |
| 47 | Ehuru | Lanlate | Ibarapa East | Oyo | Landrace |
| 48 | Ekpe1 | Ilushi | Akoko Edo | Edo | Landrace |
| 49 | Ekpe2 |  | Ikwuano | Anambra | Landrace |
| 50 | Ekunmodo1 | Kaba | Kabba/Bubu | Kogi | Landrace |
| 51 | Ekunmodo2 |  |  |  | Landrace |
| 52 | Elefu | Idumuje Ugboko | Aniocha North | Delta | Landrace |
| 53 | Ewusu |  |  |  | Landrace |
| 54 | Fakesta1 | Gboko | Gboko | Benue | Landrace |
| 55 | Fakesta2 |  | Ikwuano | Anambra | Landrace |
| 56 | Gambari1 | AyadeEkiti | Oye | Ekiti | Landrace |
| 57 | Gambari2 |  |  |  | Landrace |
| 58 | Gbango | Kisi | Irepo | Oyo | Landrace |
| 59 | Gbangu | Gboko | Gboko | Benue | Landrace |
| 60 | Gwari | Abuja | FCT |  | Landrace |
| 61 | Hembakwase1 | Agyaragu | Lafia | Nasarawa | Landrace |
| 62 | Hembakwase2 | Gboko | Gboko | Benue | Landrace |
| 63 | Hembakwase3 |  | Ikwuano | Anambra | Landrace |
| 64 | Inekedes | Ayogwuri | Etsako West | Edo | Landrace |
| 65 | Innekka | Ayogwuri | Etsako West | Edo | Landrace |
| 66 | Kampee | Kaba | Kabba/Bubu | Kogi | Landrace |
| 67 | Kangan | Kisi | Irepo | Oyo | Landrace |
| 68 | Keregbe |  |  |  | Landrace |
| 69 | Kerege | Kisi | Irepo | Oyo | Landrace |
| 70 | Kokorogbaro | IleshaBaruba | Baruten | Oyo | Landrace |
| 71 | Kwashie | Garatu | Bosso | Niger | Landrace |
| 72 | Lanshie | Garatu | Bosso | Niger | Landrace |
| 73 | Lansinrin | Lanlate | Ibarapa East | Oyo | Landrace |
| 74 | Meccakusa | Zakibiam | Ukum | Benue | Landrace |
| 75 | Myiamiyo |  | Ikwuano | Anambra | Landrace |
| 76 | Ogoja | Gboko | Gboko | Benue | Landrace |
| 77 | Sepotele | Kaba | Kabba/Bubu | Kogi | Landrace |
| 78 | Sogbo | IyeEkiti | Ilejemje | Ekiti | Landrace |
| 79 | Talamba | Igboho | Orilope | Oyo | Landrace |
| 80 | Ukpade | Ayogwuri | Etsako West | Edo | Landrace |
| 81 | Utsuakpe |  | Ikwuano | Anambra | Landrace |
| 82 | Yamgbede | Gunu | Shiroro | Niger | Landrace |
| 83 | Yandu |  | Ikwuano | Anambra | Landrace |
| 84 | Iki |  |  |  | Landrace |
| 85 | Iledu1 |  |  |  | Landrace |
| 86 | Ledu2 |  |  |  | Landrace |
| 1 | TDr1401593 |  |  |  | IITA breeding lines |
| 2 | TDr9519177 |  |  |  | IITA breeding lines |
| 3 | TDr1000021 |  |  |  | IITA breeding lines |
| 4 | TDr1400537 |  |  |  | IITA breeding lines |
| 5 | TDr1100055 |  |  |  | IITA breeding lines |
| 6 | TDr1401161 |  |  |  | IITA breeding lines |
| 7 | TDr1400158 |  |  |  | IITA breeding lines |
| 8 | TDr1401220 |  |  |  | IITA breeding lines |
| 9 | TDr0900135 |  |  |  | IITA breeding lines |
| 10 | TDr1400359 |  |  |  | IITA breeding lines |
| 11 | TDr0900295 |  |  |  | IITA breeding lines |
| 12 | TDr1400766 |  |  |  | IITA breeding lines |
| 13 | TDr1100180 |  |  |  | IITA breeding lines |
| 14 | TDr8902665 |  |  |  | IITA breeding lines |
| 15 | TDr1401419 |  |  |  | IITA breeding lines |
| 16 | TDr1100128 |  |  |  | IITA breeding lines |

^ɠ^LGA=Local Government Area

SUPPLEMENTARY TABLE 2 **|** **Agro-morphological performance of the farmers’ landraces and IITA elite yam breeding lines.**

|  | **Breeding lines** | | | |  | **Farmer varieties** | | | |
| --- | --- | --- | --- | --- | --- | --- | --- | --- | --- |
| Traits | **Min** | **Max** | **Mean** | **SD** |  | **Min** | **Max** | **Mean** | **SD** |
| DM (%) | 25.46 | 33.75 | 29.95 | 2.82 |  | 27.71 | 47.22 | 35.07 | 4.28 |
| Yield (t ha^-1^) | 8.83 | 19.16 | 13.12 | 2.72 |  | 4.57 | 36.88 | 18.25 | 8.48 |
| YMV | 221.99 | 295.06 | 250.90 | 18.86 |  | 144.08 | 381.00 | 257.37 | 36.77 |
| OXID | 1.00 | 3.00 | 2.19 | 0.66 |  | 0.00 | 2.00 | 0.19 | 0.43 |
| Vigor | 2.44 | 3.00 | 2.70 | 0.19 |  | 1.80 | 3.02 | 2.51 | 0.37 |

DM – tuber dry matter content, YMV – yam mosaic virus severity, OXID – tuber flesh oxidation, SD – standard deviation.

SUPPLEMENTARY TABLE 3 **|** **Breeding values for each trait and the index FAI-BLUP values of all the 86 yam landraces.**

| **clones** | **YMV** | **Yield** | **Vigor** | **Oxi** | **DM** | **FAI-BLUP** |
| --- | --- | --- | --- | --- | --- | --- |
| Agboghocha | 3.2012 | -2.0433 | 0.0589 | -0.0003 | -0.0416 | 4.3199 |
| Agin | 13.3201 | 4.0971 | 0.3933 | 0.0016 | -3.3209 | 4.0912 |
| Agunmaga | 1.4439 | -1.9317 | -0.0084 | -0.0003 | 1.368 | 5.9222 |
| Ahole | 21.4572 | -4.4563 | -0.0423 | 0.0012 | 2.2926 | 4.0811 |
| Ajimokunu | -44.7987 | -10.4332 | -0.2095 | -0.0002 | 4.6492 | 4.0099 |
| Akoko | -45.3593 | -9.7528 | -0.3402 | -0.0002 | 2.1666 | 4.3309 |
| Alaka | 3.6018 | 0.539 | 0.2882 | -0.0001 | -2.4063 | 3.9957 |
| Alibu | -10.9314 | 1.4543 | 0.2307 | -0.0005 | -1.4033 | 3.5370 |
| **Aloshie** | **-14.5323** | **7.6683** | **0.3185** | **-0.0003** | **-1.0313** | **2.8686** |
| Alumaco | -22.5265 | -1.2386 | -0.3437 | -0.0003 | 1.2417 | 4.2198 |
| Ameh | -8.7262 | 4.7726 | 0.2613 | -0.0002 | 2.8536 | 4.5958 |
| Ami1 | -5.9743 | 1.9621 | 0.2251 | -0.0002 | 2.7584 | 4.0177 |
| Ami2 | -15.9629 | -6.469 | -0.1352 | -0.0002 | 0.1048 | 7.1944 |
| **Amola** | **-11.007** | **-12.1343** | **-0.4441** | **0.0001** | **3.3308** | **3.1926** |
| Amole | -24.1599 | -5.7976 | 0.0074 | -0.0003 | -0.1907 | 4.0811 |
| Amula | -15.7723 | -6.705 | -0.0846 | -0.0003 | -1.3333 | 5.2444 |
| Anaswe | 7.338 | -3.1769 | 0.0465 | -0.0005 | -3.9538 | 4.1330 |
| **Anthony** | **-10.3192** | **-2.7483** | **-0.5151** | **-0.0002** | **-0.7183** | **2.7764** |
| **Anyamayowa1** | **-6.5683** | **1.7413** | **0.0587** | **-0.0003** | **2.0094** | **2.9153** |
| **Anyamayowa2** | **3.5292** | **1.3952** | **-0.2329** | **-0.0004** | **2.2053** | **3.1843** |
| **Aro** | **20.0855** | **-8.6317** | **-0.3084** | **-0.0004** | **1.0988** | **2.8498** |
| Awada | 15.4811 | 7.7068 | 0.0725 | 0.0016 | -4.0326 | 6.3748 |
| Awanoba | -3.1043 | 4.5222 | 0.1805 | -0.0004 | 4.7519 | 3.7789 |
| Boki | 31.9197 | 1.3158 | 0.314 | 0.0016 | -3.4014 | 3.4891 |
| Cheyol | -12.9583 | -1.5243 | -0.0603 | -0.0001 | 3.9984 | 3.8420 |
| Dariboko | -2.5928 | -8.0038 | -0.1947 | -0.0002 | 3.717 | 4.1938 |
| Didiya | 2.1653 | -8.0224 | -0.3586 | 0.0018 | 1.5329 | 3.8987 |
| Efunyebe | 4.1475 | -2.7766 | -0.269 | -0.0002 | -0.348 | 3.8672 |
| Ehara | 1.8808 | 11.7144 | 0.3168 | -0.0003 | 0.0229 | 4.0857 |
| **Ehuru** | **-5.8061** | **9.3633** | **0.2147** | **-0.0003** | **1.3053** | **3.1554** |
| Ekpe1 | 12.7455 | 0.243 | 0.1169 | -0.0003 | 1.2226 | 3.7072 |
| **Ekpe2** | **-11.3109** | **-4.3412** | **-0.112** | **-0.0003** | **0.749** | **3.0803** |
| Ekunmodo1 | 23.0731 | -1.2006 | -0.0722 | 0.0012 | -2.2295 | 3.8309 |
| Ekunmodo2 | 11.2547 | -0.9231 | -0.3502 | -0.0005 | 2.4758 | 4.0811 |
| Elefu | 18.691 | 8.2752 | -0.0331 | -0.0005 | 0.4912 | 3.8851 |
| **Ewusu** | **13.1868** | **3.5974** | **-0.0745** | **0.0013** | **-2.3144** | **3.3672** |
| Fakesta1 | 15.8366 | -0.3951 | 0.0964 | -0.0004 | 0.0394 | 3.3691 |
| Fakesta2 | 10.0727 | 9.2249 | 0.2939 | -0.0003 | -1.7915 | 3.2744 |
| Gambari1 | -9.304 | -5.0308 | -0.1429 | -0.0003 | -2.8855 | 5.8403 |
| Gambari2 | 23.101 | 7.5483 | -0.0527 | 0.0012 | -1.0163 | 4.1979 |
| Gbango | 13.0964 | 1.7392 | 0.0873 | -0.0003 | -1.8309 | 4.6121 |
| Gbangu | 12.297 | 1.064 | 0.0649 | -0.0003 | 5.2561 | 3.9693 |
| Gwari | 11.082 | 4.5297 | -0.0174 | -0.0001 | 2.0442 | 3.7066 |
| Hembakwase1 | 8.116 | 10.6531 | 0.1297 | -0.0001 | -1.0325 | 3.7389 |
| Hembakwase2 | 8.2063 | -4.4401 | 0.0557 | -0.0004 | 0.113 | 4.2096 |
| Hembakwase3 | 10.5582 | -4.9503 | 0.0209 | -0.0001 | -1.1912 | 3.4749 |
| **Iki** | **-3.0848** | **-5.0661** | **-0.2262** | **-0.0002** | **5.4435** | **2.8670** |
| **Iledu** | **-17.528** | **-6.9017** | **-0.1877** | **-0.0002** | **-1.7717** | **3.3254** |
| Inekedes | 0.9522 | 3.2231 | 0.1847 | -0.0003 | 0.6103 | 4.4927 |
| Innekka | 5.9748 | 3.1625 | 0.1157 | -0.0004 | 0.3897 | 6.7922 |
| Inodu | -18.8087 | 0.9001 | 0.2442 | -0.0005 | -0.1962 | 4.6584 |
| **Iphara** | **-5.2605** | **-1.5503** | **0.2313** | **-0.0005** | **-5.2349** | **3.3261** |
| Kampee | 10.9066 | 8.5688 | 0.2268 | -0.0001 | -0.2203 | 5.0368 |
| Kangan | 6.0225 | 3.2864 | 0.1297 | -0.0002 | -1.1434 | 4.5927 |
| Keregbe | 19.016 | 0.5891 | 0.0022 | 0.0005 | -4.4374 | 3.7779 |
| Kerege | -18.9959 | -5.0324 | -0.4216 | -0.0002 | 0.8133 | 3.8008 |
| Kioyo | -25.5655 | -0.9349 | 0.2048 | -0.0004 | -3.8088 | 4.7151 |
| Kogi | 1.6891 | 9.2576 | 0.1408 | -0.0005 | -3.5497 | 3.5238 |
| Kokorogbaro | -11.0768 | 2.2722 | 0.0753 | -0.0003 | 3.9878 | 4.3657 |
| Kokumo | -40.056 | 0.9175 | 0.0648 | 0.0007 | -3.2464 | 4.2320 |
| Kwashie | -1.929 | -9.3947 | -0.31 | -0.0002 | -3.8543 | 4.2772 |
| Lanshie | 19.5227 | 3.4553 | 0.3702 | -0.0002 | -2.6151 | 4.8547 |
| Lansinrin | 10.0648 | 9.8346 | 0.3131 | -0.0001 | 0.8781 | 4.6660 |
| Ledu | -30.056 | 0.9175 | 0.0648 | 0.0007 | -3.2464 | 3.8897 |
| Meccakusa | 12.2181 | 2.684 | 0.1378 | 0.0005 | -4.2646 | 4.5193 |
| Myiamiyo | 41.196 | 12.3492 | 0.3348 | -0.0003 | -4.0552 | 4.0839 |
| Ngwoka | 16.4811 | 7.7068 | 0.0725 | 0.0016 | -4.0326 | 4.7151 |
| Nndu1 | -13.0949 | 1.5712 | 0.0043 | 0.0019 | 1.4056 | 4.7151 |
| Nndu2 | -12.0949 | 1.5712 | 0.0043 | 0.0019 | 1.4056 | 4.5179 |
| Nwopoko | -17.1341 | -6.1521 | -0.4061 | 0.0018 | 3.9035 | 5.2910 |
| Obiaturugo | 14.3201 | 4.0971 | 0.3933 | 0.0016 | -3.3209 | 4.4118 |
| Ogoja1 | 22.4572 | -4.4563 | -0.0423 | 0.0012 | 2.2926 | 5.4369 |
| Ogoja2 | -11.3192 | -2.7483 | -0.5151 | -0.0002 | -0.7183 | 5.1604 |
| Pepa1 | 26.0731 | -1.2006 | -0.0722 | 0.0012 | -2.2295 | 5.2527 |
| Pepa2 | 15.1868 | 3.5974 | -0.0745 | 0.0013 | -2.3144 | 4.9293 |
| Pepa3 | -18.1341 | -6.1521 | -0.4061 | 0.0018 | 3.9035 | 5.5771 |
| Peupeu | 29.101 | 7.5483 | -0.0527 | 0.0012 | -1.0163 | 3.7779 |
| Punch1 | 20.016 | 0.5891 | 0.0022 | 0.0005 | -4.4374 | 4.6448 |
| Punch2 | 14.2181 | 2.684 | 0.1378 | 0.0005 | -4.2646 | 3.6401 |
| Sepotele | -1.5952 | -4.5853 | -0.2585 | -0.0001 | 0.6195 | 8.1177 |
| Sogbo | -2.6075 | -11.4538 | -0.4211 | -0.0001 | 3.0214 | 6.1267 |
| Talamba | -13.007 | -12.1343 | -0.4441 | 0.0001 | 3.3308 | 3.8231 |
| Ukpade | -1.5952 | -4.5853 | -0.2585 | -0.0001 | 0.6195 | 6.7095 |
| Utsuakpe | -3.6075 | -10.4538 | -0.4211 | -0.0001 | 3.0214 | 4.0779 |
| **Yamgbede** | **-4.0848** | **-4.0661** | **-0.2262** | **-0.0002** | **5.4435** | **2.9977** |
| Yandu | -17.528 | -7.9017 | -0.1877 | -0.0002 | -1.7717 | 5.4552 |

YMV: yam mosaic virus severity; DM: dry matter content. Rows in bold are for the landraces with low FAI-BLUP (<3.5). The bold red font shows the 13 top ranking high multi-trait performing yam landraces based on FAI-BLUP ideotype-design.

**SUPPLEMENTARY TABLE 4: List of 86 yam landraces with their admixture groups, hierarchical clusters and their levels of heterozygosity.**

| **S/N** | **Landraces** | **Admixture group** | **HC^ɠ^** | **HeLe^ɠ^** |
| --- | --- | --- | --- | --- |
| 1 | Alibu | 1 | 1 | 1 |
| 2 | Anaswe | 1 | 1 | 1 |
| 3 | Awada | 1 | 1 | 1 |
| 4 | Inodu | 1 | 1 | 1 |
| 5 | Iphara | 1 | 1 | 1 |
| 6 | Kioyo | 1 | 1 | 1 |
| 7 | Kogi | 1 | 1 | 1 |
| 8 | Didiya | 2 | 2 | 0.02876 |
| 9 | Nndu1 | 2 | 2 | 0.04292 |
| 10 | Nndu2 | 2 | 2 | 0.04943 |
| 11 | Nwopoko | 2 | 2 | 0.04876 |
| 12 | Obiaturugo | 2 | 2 | 0.08815 |
| 13 | Ogoja1 | 2 | 2 | 0.1973 |
| 14 | Pepa1 | 2 | 2 | 0.26313 |
| 15 | Pepa2 | 2 | 2 | 0.26313 |
| 16 | Pepa3 | 2 | 2 | 0.26313 |
| 17 | Peupeu | 2 | 2 | 0.26287 |
| 18 | Ngwoka | 2 | 2 | 0.12256 |
| 19 | Kokumo | Admixture | 2 | 0.33042 |
| 20 | Punch1 | Admixture | 2 | 0.26404 |
| 21 | Punch2 | Admixture | 2 | 0.26404 |
| 22 | Amole | 2 | 3 | 0.02727 |
| 23 | Agboghocha | 3 | 3 | 0.05327 |
| 24 | Agin | 3 | 3 | 0.07156 |
| 25 | Agunmaga | 3 | 3 | 0.04254 |
| 26 | Ahole | 3 | 3 | 0.05726 |
| 27 | Ajimokunu | 3 | 3 | 0.05844 |
| 28 | Akoko | 3 | 3 | 0.05845 |
| 29 | Alaka | 3 | 3 | 0.08508 |
| 30 | Aloshie | 3 | 3 | 0.04313 |
| 31 | Alumaco | 3 | 3 | 0.05578 |
| 32 | Ameh | 3 | 3 | 0.04551 |
| 33 | Ami1 | 3 | 3 | 0.04551 |
| 34 | Ami2 | 3 | 3 | 0.04575 |
| 35 | Amola | 3 | 3 | 0.04499 |
| 36 | Amula | 3 | 3 | 0.04499 |
| 37 | Anthony | 3 | 3 | 0.1224 |
| 38 | Anyamayowa1 | 3 | 3 | 0.03681 |
| 39 | Anyamayowa2 | 3 | 3 | 0.07782 |
| 40 | Aro | 3 | 3 | 0.04888 |
| 41 | Awanoba | 3 | 3 | 0.04188 |
| 42 | Boki | 3 | 3 | 0.21791 |
| 43 | Cheyol | 3 | 3 | 0.1259 |
| 44 | Dariboko | 3 | 3 | 0.11204 |
| 45 | Efunyebe | 3 | 3 | 0.03256 |
| 46 | Ehara | 3 | 3 | 0.04129 |
| 47 | Ehuru | 3 | 3 | 0.05199 |
| 48 | Ekpe1 | 3 | 3 | 0.05356 |
| 49 | Ekpe2 | 3 | 3 | 0.05356 |
| 50 | Ekunmodo1 | 3 | 3 | 0.06859 |
| 51 | Ekunmodo2 | 3 | 3 | 0.07261 |
| 52 | Elefu | 3 | 3 | 0.07154 |
| 53 | Ewusu | 3 | 3 | 0.0697 |
| 54 | Fakesta1 | 3 | 3 | 0.05202 |
| 55 | Fakesta2 | 3 | 3 | 0.05518 |
| 56 | Gambari1 | 3 | 3 | 0.08729 |
| 57 | Gambari2 | 3 | 3 | 0.04065 |
| 58 | Gbango | 3 | 3 | 0.04617 |
| 59 | Gbangu | 3 | 3 | 0.03967 |
| 60 | Gwari | 3 | 3 | 0.08729 |
| 61 | Hembakwase1 | 3 | 3 | 0.08508 |
| 62 | Hembakwase2 | 3 | 3 | 0.05202 |
| 63 | Hembakwase3 | 3 | 3 | 0.06508 |
| 64 | Inekedes | 3 | 3 | 0.05202 |
| 65 | Innekka | 3 | 3 | 0.05202 |
| 66 | Kampee | 3 | 3 | 0.08508 |
| 67 | Kangan | 3 | 3 | 0.04081 |
| 68 | Keregbe | 3 | 3 | 0.0644 |
| 69 | Kerege | 3 | 3 | 0.0844 |
| 70 | Kokorogbaro | 3 | 3 | 0.0575 |
| 71 | Kwashie | 3 | 3 | 0.04697 |
| 72 | Lanshie | 3 | 3 | 0.04983 |
| 73 | Lansinrin | 3 | 3 | 0.08508 |
| 74 | Meccakusa | 3 | 3 | 0.06341 |
| 75 | Myiamiyo | 3 | 3 | 0.04377 |
| 76 | Ogoja2 | 3 | 3 | 0.09405 |
| 77 | Sepotele | 3 | 3 | 0.07922 |
| 78 | Sogbo | 3 | 3 | 0.11036 |
| 79 | Talamba | 3 | 3 | 0.1258 |
| 80 | Ukpade | 3 | 3 | 0.06137 |
| 81 | Utsuakpe | 3 | 3 | 0.0475 |
| 82 | Yamgbede | 3 | 3 | 0.04937 |
| 83 | Yandu | 3 | 3 | 0.05067 |
| 84 | Iki | Admixture | 3 | 0.04171 |
| 85 | Iledu | Admixture | 3 | 0.03695 |
| 86 | Ledu | Admixture | 3 | 0.02791 |

^ɠ^*HC: Hierarchical clustering, HeLe: Heterozygosity level*

SUPPLEMENTARY TABLE 5 | **Genetic variability among and within different Nigeria states**

| **State** | Ananbra | Benue | Delta | Edo | Ekiti | FCT | Kogi | Kwara | Nassarawa | Niger | Oyo |
| --- | --- | --- | --- | --- | --- | --- | --- | --- | --- | --- | --- |
| Ananbra | -0.043 |  |  |  |  |  |  |  |  |  |  |
| Benue | -0.014 | -0.071 |  |  |  |  |  |  |  |  |  |
| Delta | 0.225 | 0.082 | -0.280 |  |  |  |  |  |  |  |  |
| Edo | -0.012 | -0.059 | 0.073 | -0.076 |  |  |  |  |  |  |  |
| Ekiti | -0.098 | -0.051 | 0.119 | -0.042 | -0.266 |  |  |  |  |  |  |
| FCT | -0.405 | -0.341 | 0.003 | -0.399 | -0.396 | - |  |  |  |  |  |
| Kogi | 0.019 | 0.051 | 0.433 | 0.041 | 0.177 | 0.016 | -0.072 |  |  |  |  |
| Kwara | -0.405 | -0.360 | -0.011 | -0.423 | -0.229 | - | -0.114 | - |  |  |  |
| Nassarawa | -0.117 | -0.073 | 0.178 | -0.067 | -0.178 | -0.420 | 0.109 | -0.420 | -0.283 |  |  |
| Niger | -0.068 | -0.028 | 0.226 | -0.037 | -0.110 | -0.583 | 0.029 | -0.613 | -0.159 | -0.194 |  |
| Oyo | -0.020 | 0.030 | 0.322 | 0.026 | -0.048 | -0.340 | 0.026 | -0.348 | -0.081 | -0.073 | -0.052 |

Fixation index in the diagonal represent the Fst value within states while the lower value are the Fst among the states

SUPPLEMENTARY TABLE 6 | **Factor loading displaying variables associated with the three discriminate factors and predicted genetic gain based on the selection for FAI-BLUP index.**

| **Trait** | **FA1** | **FA2** | **FA3** | **Sense** | **Communality** | **Uniqueness** | **Predicted genetic gain (%)** |
| --- | --- | --- | --- | --- | --- | --- | --- |
| YMV | **0.561** | 0.485 | 0.451 | Decrease | 0.753 | 0.247 | -5.72 |
| OXBI | 0.200 | 0.161 | **-0.881** | Decrease | 0.843 | 0.157 | 0.554 |
| Vigor | **0.713** | 0.0287 | -0.0962 | Increase | 0.518 | 0.482 | 7.48 |
| Yield | **0.807** | -0.286 | -0.149 | Increase | 0.755 | 0.245 | 3.92 |
| DMC | -0.154 | **0.887** | -0.176 | Increase | 0.841 | 0.159 | -164 |
| **Eigen value** | 1.55 | 1.15 | 1.01 |  |  |  |  |
| **Contribution** | 31.1 | 22.9 | 20.2 |  |  |  |  |
| **Cumulative** | 31.1 | 54.0 | 74.2 |  |  |  |  |

 YMV: Yam mosaic virus, DMC: Dry matter content, OXBI: Oxidative browning index

SUPPLEMENTARY TABLE 7 | **The number of cross combinations predicted per landrace and its average crossing merit from the different cross combinations**.

| **Landraces** | **Sex** | **Number of cross combinations** | **Average crossing merit** |
| --- | --- | --- | --- |
| Bokipepa | Female | 31 | -0.06 |
| Yandu | Female | 31 | 0.29 |
| Meccakusa | Female | 27 | -0.44 |
| Pepa1 | Female | 26 | 0.32 |
| Peupeu | Female | 25 | 0.65 |
| **Anyamayowa2** | **Female** | **24** | **1.22** |
| Ameh | Female | 22 | 0.67 |
| **Anyamayowa1** | **Female** | **22** | **1.02** |
| Cheyol | Female | 22 | 1.29 |
| **Aloshie** | **Female** | **19** | **-0.07** |
| Ami1 | Female | 19 | 1.31 |
| Awanoba | Female | 19 | 1.03 |
| Pepa2 | Female | 19 | -0.48 |
| **Iphara** | **Female** | **18** | **0.01** |
| Kampee | Female | 18 | -0.97 |
| Hembakwase1 | Female | 17 | 0.45 |
| Ngwoka | Female | 17 | 0.63 |
| Nndu1 | Female | 17 | -0.44 |
| Nndu2 | Female | 17 | 0.56 |
| Pepa3 | Female | 15 | 1.69 |
| Alaka | Female | 14 | 1.68 |
| **Ekpe2** | **Female** | **13** | **1.03** |
| Lansinrin | Female | 13 | 0.93 |
| Hembakwase2 | Female | 11 | -0.4 |
| Hembakwase3 | Female | 8 | -1.1 |
| **Iledu** | **Male** | **21** | **0.99** |
| Didiya | Male | 20 | 0.14 |
| **Amola** | **Male** | **19** | **0.74** |
| Ekunmodu | Male | 19 | -0.21 |
| Kioyo | Male | 19 | -0.89 |
| **Anthony** | **Male** | **18** | **-0.44** |
| Punch2 | Male | 17 | 1.64 |
| Agin | Male | 16 | 1.64 |
| **Aro** | **Male** | **15** | **-0.05** |
| Awada | Male | 15 | 0.24 |
| **Ewusu** | **Male** | **15** | **0.54** |
| Fakesta1 | Male | 15 | -0.66 |
| Sepotele | Male | 15 | 0.29 |
| Ahole | Male | 14 | -0.38 |
| Kokorogbaro | Male | 14 | 1.03 |
| Innekka | Male | 14 | 0.81 |
| Sogbo | Male | 14 | -0.89 |
| Ami2 | Male | 13 | 0.38 |
| Gambari2 | Male | 13 | 0.59 |
| Kwashie | Male | 13 | -0.81 |
| Amole | Male | 12 | -0.75 |
| Anaswe | Male | 12 | 0.86 |
| Inodu | Male | 12 | 1.16 |
| Keregbe | Male | 12 | 0.24 |
| Akoko | Male | 11 | -0.59 |
| **Iki** | **Male** | **11** | **1.26** |
| **Yamgbede** | **male** | **11** | **-0.22** |
| **Ehuru** | **Male** | **10** | **-1.32** |
| Ledu | Male | 10 | 0.16 |
| Ajimokunu | Male | 9 | -1.11 |
| Ehara | Male | 9 | 0.76 |
| Inekedes | Male | 8 | -0.62 |
| Ukpade | Male | 8 | 1.31 |
| Utsuakpe | Male | 8 | 0.46 |
| Fakesta2 | Male | 6 | -0.2 |
| Kogi | male | 5 | 1.79 |

**Bold** indicates those landraces identified as top multi-trait performing based on FAI-BLUP index
